# Antibiotic type and dose variably affect microbiomes of a disease-resistant *Acropora cervicornis* genotype

**DOI:** 10.1186/s40793-025-00709-2

**Published:** 2025-05-02

**Authors:** Sunni Patton, Denise P. Silva, Eddie Fuques, Grace Klinges, Erinn M. Muller, Rebecca L. Vega Thurber

**Affiliations:** 1 Ecology, Evolution, and Marine Biology, University of California, Santa Barbara, Santa Barbara, CA 93106-9620 USA; 2 Department of Microbiology, Oregon State University, 226 Nash Hall, Corvallis, OR 97331 USA; 3 Center for Global Discovery and Conservation Science, Arizona State University, Hilo, HI 96720 USA; 4 Mote Marine Laboratory, 1600 Ken Thompson Pkwy, Sarasota, FL 34236 USA; 5 Mote Marine Laboratory International Center for Coral Reef Research and Restoration, 24244 Overseas Hwy, Summerland Key, FL 33042 USA

**Keywords:** *Acropora cervicornis*, Antibiotics, Disease-resistance, Microbiome

## Abstract

**Background:**

As coral diseases become more prevalent and frequent, the need for new intervention strategies also increases to counteract the rapid spread of disease. Recent advances in coral disease mitigation have resulted in increased use of antibiotics on reefs, as their application may halt disease lesion progression. Although efficacious, consequences of deliberate microbiome manipulation resulting from antibiotic administration are less well-understood– especially in non-diseased corals that appear visually healthy. Therefore, to understand how apparently healthy corals are affected by antibiotics, we investigated how three individual antibiotics, and a mixture of the three, impact the microbiome structure and diversity of a disease-resistant Caribbean staghorn coral (*Acropora cervicornis*) genotype. Over a 96-hour, aquarium-based antibiotic exposure experiment, we collected and processed coral tissue and water samples for 16S rRNA gene analysis.

**Results:**

We found that antibiotic type and dose distinctively impact microbiome alpha diversity, beta diversity, and community composition. In experimental controls, microbiome composition was dominated by an unclassified bacterial taxon from the order *Campylobacterales*, while each antibiotic treatment significantly reduced the relative abundance of this taxon. Those taxa that persisted following antibiotic treatment largely differed by antibiotic type and dose, thereby indicating that antibiotic treatment may result in varying potential for opportunist establishment.

**Conclusion:**

Together, these data suggest that antibiotics induce microbiome dysbiosis– hallmarked by the loss of a dominant bacterium and the increase in taxa associated with coral stress responses. Understanding the off-target consequences of antibiotic administration is critical not only for informed, long-term coral restoration practices, but also for highlighting the importance of responsible antibiotic dissemination into natural environments.

**Supplementary Information:**

The online version contains supplementary material available at 10.1186/s40793-025-00709-2.

## Background

Host-associated microorganisms often confer benefits that augment host development and physiology, protect against pathogen infection, or provide other desirable fitness advantages such as feeding adaptations and phenotypic plasticity [1–7]. Microbiome structure and influence are not unilateral, however, and intricate host-microbe-environment interactions each contribute to fitness, stable microbiome structure, and cohesion between the host and host-associated microbial counterparts (recognized as the holobiont) [8–10]. While the resident microbiota of healthy hosts often provides an additional buffer against minor disturbances and environmental fluctuations [11, 12], major disruptions to these relationships often support opportunist invasion, infection, and/or microbiome dysbiosis. Across systems, microbiome dysbiosis is thought to be an indicator of imbalance between beneficial and harmful bacteria and, depending on the disturbance, can be hallmarked by increased stochastic dispersion and reduced diversity [13–15]. Given the range of potential outcomes, it is essential to understand how host-associated microbiomes facilitate disturbance resistance and resilience, and how alterations in microbe-host and microbe-microbe associations may compromise holobiont synergy– especially in sensitive or endangered species.

Sensitive ecosystems such as coral reefs are consistently and increasingly threatened by a suite of local and global environmental perturbations, resulting in holobiont disturbances that jeopardize coral survivability and the biodiverse landscapes and resources they provide. In addition to supporting high macrodiversity, corals also provide a complex environment for microbial life, as distinct microhabitats within the coral (i.e., surface mucus layer, tissue, and skeleton) support diverse and localized bacterial community structures [16, 17]. These compartments vary in microbial abundance, diversity, and susceptibility to environmental disturbance– with the surface mucus layer providing a level of defense against disturbances, as it is at the coral-environment interface [12, 18]. Moreover, while some corals acquire microbiome members vertically, many acquire a substantial portion of their microbiome horizontally [19], further emphasizing the influence that environmental conditions have on coral microbiomes.

These environmental threats include an array of biotic and abiotic factors such as thermal anomalies, nutrient pollution, sedimentation, and macroalgal overgrowth [20–24]. Many of these environmental stressors are also not mutually exclusive and often act synergistically with one another to further exacerbate coral health decline through microbiome dysbiosis and subsequent disease [25–30], although some evidence suggests stressors can sometimes act antagonistically as well [31]. Since the first coral diseases were documented in the 1970s, approximately 40 diseases have been described, with only six having known etiological agents [10, 32, 33]. In many cases, validating a causative agent via Koch’s postulates– which requires pure isolation and cultivation of the suspected pathogen and subsequent reinfection– is difficult due to the obvious differences between laboratory and reef conditions, incomplete understanding of transmission dynamics, as well as culturing limitations [34–36]. Additionally, many coral diseases are likely not caused by a single pathogen, as several are considered polymicrobial or the result of a secondary infection [37–40].

Disentangling coral disease is a complex task, and several studies have attempted to identify a core coral microbiome to understand which microbial members are expected, or a deviation due to disturbance [41, 42]. Some core members include the endosymbiotic taxa *Actinomycelates* and *Burkholderiales*, yet uncovering a unifying core structure is likely still dependent on a combination of coral species, geographic location, life history, body site sampled, and parameters used to identify these core taxa. Additionally, given the complex association of coral microorganisms, many taxa linked to disease are also found in healthy individuals, further obscuring disease etiology [43–45].

Owing to the array of diseases with undetermined causes, and difficult diagnostic methods, restoration efforts largely focus on immediate disease remediation. Two highly transmissible diseases at the forefront of disease remediation efforts are Stony Coral Tissue Loss Disease (SCTLD) and White Band Disease (WBD). First characterized in Florida in 2014, SCTLD is hallmarked by unique disease epidemiology due to its wide geographic and host range and rapid disease progression, which often results in mortality [46–48]. This disease affects more than 20 coral species throughout the Atlantic– many of which are considered endangered by the International Union for Conservation of Nature Red List [46, 49]. Despite the broad species range of SCTLD, this disease has not yet been identified in *Acropora* corals such as *A. cervicornis* and *A. palmata.* These species are instead plagued by WBD, which has been responsible for the large-scale population mortality since the late 1970s [50]. Currently, there are no definitive causes of this disease either, although it is thought to be bacterial [51]. This disease also results in visible lesions and widespread, rapid mortality– although the host range is far smaller than that of SCTLD as it only currently affects *Acropora cervicornis* and *Acropora palmata* [50].

To control the spread of diseases, recent efforts have focused on utilizing broad-spectrum antibiotics, namely ampicillin and amoxicillin pastes [52–56]. Given the widespread and elusive nature of these diseases, antibiotics are being utilized not only as a way to confirm a bacterial component of the disease, but also to provide an immediate solution while the etiological agent(s) remain unidentified. Amoxicillin pastes have demonstrated clear efficacy against SCTLD in short (~ 2 weeks) and long-term (~ 1 year) in-situ and ex-situ experiments, although treatment efficacy may be dependent on coral species– likely due, in part, to morphological characteristics not conducive to antibiotic application [52, 54–56], as well as burgeoning antibiotic resistance within the pathogen(s). Treatment regimens differ across studies, yet each consistently reports that amoxicillin pastes slow or halt disease lesion progression into a quiescent state [55, 56]. Despite the greater than 90% success rate in many cases, these intervention strategies are likely only a temporary solution as antibiotic administration does not prevent new lesion formation [54–56]. This may indicate that the current antibiotic does not target the true causative agent, that retreatment strategies require further optimization, or that the waterborne pathogen(s) are transmitted to other parts of the coral colony prior to treatment intervention.

In addition to the ecotoxicological and antibiotic resistance concerns of antibiotic contamination in reef systems, several studies have noted that antibiotic-induced disruption of coral microbiomes results in a diminished capacity to withstand subsequent stressors such as heat stress and transplantation to a natural system [12, 57–59]. Evidence suggests, specifically within the genus *Pocillopora*, that disruptions to the holobiont can cause increased transcriptional stress responses from both the coral host and algal symbiont, as well as decreased bacterial diversity which, when combined, reduces heat tolerance and upregulates immune response genes [58, 60]. Therefore, with the threat of subsequent and concurrent disturbances, it is imperative to pair microbiome studies with antibiotic interventions to understand how both target and non-target microbiomes are affected.

In this study, we investigated how a 96-hour exposure to one of two concentrations of ampicillin, streptomycin, ciprofloxacin, and a mixture of the three, affects microbiome composition and diversity in a disease-resistant *Acropora cervicornis* genotype as a representative off-target species. Our results suggest that antibiotics reduce dominant taxa and allow for potentially harmful bacteria to proliferate. Additionally, they suggest that antibiotic dose range-finding is essential for future disease interventions, as different concentrations of the same antibiotic may result in distinct microbial community profiles.

## Methods

### Experimental design

A total of 120 coral fragments of a disease-resistant *Acropora cervicornis* genotype (ML-7) were collected from the offshore coral nursery at Mote Marine Laboratory’s International Center for Coral Reef Research and Restoration (IC2R3) in Summerland Key, Florida. Fragments were attached to ceramic plugs using cyanoacrylate glue and acclimated to ex-situ raceway conditions for 14 days in IC2R3’s Climate and Ocean Acidification Simulator (CAOS) system before experimentation. These raceway systems utilize canal water that has been degassed, filtered, foam fractionated, and UV-treated. Although the system supports temperature manipulation, the raceways were kept at 27.5 °C for the duration of the experiment. Following acclimation, coral fragments were each randomly assigned to one of nine experimental treatments: control, ‘ampicillin low’ and ‘ampicillin high’ (final tank concentrations of 10 mg/L and 100 mg/L, respectively), ‘streptomycin low’ and ‘streptomycin high’ (10 mg/L and 100 mg/L), ‘ciprofloxacin low’ and ‘ciprofloxacin high’ (due to high potency, 1 mg/L and 10 mg/L concentrations were used for low and high doses, respectively), and ‘mixture low’ and ‘mixture high’. ‘Mixture low’ and ‘mixture high’ were comprised of a combination of low or high doses of ampicillin, streptomycin, and ciprofloxacin, respectively. Fragments were then added to corresponding non-flow-through 5-gallon aquaria containing 6 L of water. Aside from the control which had a total of six tanks, each treatment had three replicate tanks with four coral fragments in each tank (i.e. n = 24 for control treatment and n = 12 for each other treatment). Each tank was then randomly distributed across three outdoor raceways. Samples were collected prior to antibiotic treatment (Time 0), and at 12, 24, 48, and 96 hours during treatment (Supplementary file 1 Figure S1). Because experimental aquaria were enclosed, tank water was manually refreshed by replacing half of the volume of water at each sampling time point and 72 hours after initial antibiotic treatment. To ensure a consistent, four-day antibiotic challenge, additional half-doses of antibiotics were added each time the water was changed, such that the final antibiotic concentration in the tanks was consistent throughout the experiment. After time 0 sampling, all four coral fragments within one of the control tanks (control 1), were sacrificed for other analyses; therefore, at each subsequent time point, only five control tanks were sampled (n = 20).

### Antibiotic preparation and dosing

Three broad-spectrum antibiotics (ampicillin, streptomycin sulfate, and ciprofloxacin anhydrous) were chosen due to their diverse mechanisms of action, bactericidal nature, and common use in antibiotic experiments on coral [58–61]. Ampicillin is a beta-lactam antibiotic that inhibits cell wall synthesis [62]; streptomycin is an aminoglycoside that interferes with protein synthesis [63]; and ciprofloxacin is a fluoroquinolone that inhibits DNA gyrase which ultimately impedes DNA replication [64]. Antibiotic working solutions were prepared using 0.2 μm filter-sterilized seawater. Once added to the experimental tanks, the final high and low concentrations of streptomycin and ampicillin were 100 µg/mL and 10 µg/mL, and 10 µg/mL and 1 µg/mL for ciprofloxacin. At each time point, control tanks were supplemented with equal volumes of the same filter-sterilized seawater that was used to make the antibiotic solutions. Antibiotic solutions were selected over an antibiotic paste to ensure even antibiotic exposure, and to not preclude physical space on the coral that could be used for sampling.

Although amoxicillin is the standard for SCTLD treatment, ampicillin was chosen due to its identical mechanism of action and its use in other coral experiments– namely in WBD-affected *Acropora cervicornis* [61]. Furthermore, these three antibiotics are often used in experiments at these high-dose concentrations [58–61], and do not appear to result in negative host phenotypes based on visual assessments of coral color and tissue health [61]. In addition to using standard experimental doses, we also included a lower threshold to explore dose-dependent responses.

### Sample collection and processing

At each time point, before sampling coral fragments, three liters of aquaria water were removed from each tank to conduct a half-tank water change– one liter of which was retained and filtered using a peristaltic pump and 0.22 μm Sterivex filter unit (model SVGP01050 Millepore Sigma) for 16 S rRNA gene analysis of the bacteria in the water column. Each Sterivex filter was placed in sterile bags (Whirl-Pak) and stored at -80 °C until processing. Avoiding the apical polyp and any previous wounds, two verrucae were snipped from each coral using sterile bone cutters and placed in a 1.2 mL cryogenic tube in 500 µL of DNA/RNA Shield (Zymo Research, Irvine, CA, USA). The samples were promptly stored at -80 °C until they were processed using the DNeasy 96 PowerSoil Pro kit (Qiagen) using the OT-2 liquid handling system (Opentrons).

### DNA extraction of water samples

Sterivex filter cartridges were sealed at one end using a Luer-Lok cap. Then 460 µL of extraction buffer solution (composed of 40 µL proteinase K, 200 µL of buffer AL provided by the Qiagen Blood & Tissue kit, and 220 µL of PBS) was added to the column. The other end was then sealed with another Luer-Lok cap, and both ends were wrapped in parafilm to avoid leakage. The filter cartridges were then attached horizontally inside of a hybridization incubator (Robbins Scientific Model 400) and incubated at 56 °C for 4 h at 20 rpm. After incubation, one end of the cartridge was uncapped and placed into a 2 mL tube and sealed with parafilm. The attached 2 mL tube was then centrifuged at 5,000 x g for 2 min inside of a 50 mL conical tube to elute the extracted DNA. After centrifugation, the 2 mL tube was stored for downstream use.

###  16S rRNA gene amplicon library preparation

The V4 region of the 16S rRNA gene from coral and seawater samples was amplified via a one-step polymerase chain reaction (PCR) approach. 25 µL PCR reactions were made using 10 µL of Platinum II *Taq* Hot-Start PCR Master Mix (2x) (Invitrogen) master mix, 2.5 µL each of 10 µM primers 515F (5’ - GTGYCAGCMGCCGCGGTAA − 3’) and 806R (5’ - GGACTACNVGGGTWTCTAAT − 3’) [65] with attached barcodes for dual-indexed libraries (for details see Silva et al., 2023 [66]). Three negative controls were included in each 96-well plate for a total of 21 negative controls. These negative controls were both DNA extraction controls (*n* = 7) and PCR negative controls (*n* = 14). The template DNA was amplified using the following thermocycler parameters: initial denaturation at 94 °C for 2 min, followed by 35 cycles of denaturation at 94 °C for 30 s, annealing at 60 °C for 30 s, and extension at 68 °C for 60 s, followed by a single final extension step at 68 °C for 10 min. Amplified PCR products were then purified using Agencourt AMPure XP beads (Beckman Coulter) following the manufacturer’s guidelines. However, 80% ethanol was used for the washing steps, and a 5-minute drying step was included after the final ethanol wash to evaporate excess ethanol. After purification, DNA concentrations were quantified using the BioTek Synergy H1 multi-mode plate reader. Libraries were pooled at equimolar concentrations before paired-end 2 × 300 bp sequencing using the Illumina NextSeq 2000 P1 system at Oregon State University’s Center for Quantitative Life Science (CQLS).

### Raw read quality control and sequence preprocessing

A total of 602 samples (21 of which were negative controls) were demultiplexed, and individual forward and reverse quality profiles were assessed using FastQC and MultiQC [67]. Primer sequences were removed from forward and reverse reads using a two-step *cutadapt* approach to remove forward and reverse primer sequences, as well as their reverse complements [68]. Reads were imported into RStudio (v. 4.3.0) for subsequent quality control processing. Using DADA2 (v. 1.28.0) [69], low-quality sequences were filtered by truncating the reads at the 3’ end at 245 bp and 230 bp for the forward and reverse reads, respectively, based on MultiQC reports.

To account for the large number of samples and reads, we used five times the number of bases to estimate the sequencing error than the default. Sample sequence identity was then inferred by DADA2::*dada* using default parameters. Then, only those contigs within the amplicon size target range (251–255 bp for coral samples and 253–254 bp for seawater samples), and determined as non-chimeric, were used in further analyses. Taxonomy was assigned down to the species level, when possible, using the SILVA nr 99 v138.1 and the SILVA Species Assignment v138.1 training set [70]. Those sequences identified as chloroplast or mitochondria, or those that were not annotated beyond the Kingdom level, were excluded.

A phyloseq object was then created using the *phyloseq* package (v. 1.44.0) in R [71]. Through the ‘combined detection method’ in the *decontam* package (v. 1.20.0; Supplementary file 1), contaminants were identified on the basis of both prevalence and quantification thresholds from negative control samples and were subsequently removed from all samples [72]. After contaminant ASVs were removed, negative controls were excluded from downstream analysis. Due to high experimental replication, taxa that had both low frequency (appearing in only one sample) and low abundance (reads within the first quartile of read distribution) were removed. Samples with fewer than 1000 reads after all quality control filtering (8) were removed from the analysis. After filtering, 5,660,802 reads and 2,962 ASVs across 573 coral samples remained.

Antibiotic treatment had a significant effect on library size (i.e., number of reads) across treatment groups at each time point except for T0 (*p* = 2.06e^− 7^, *p* = 6.92e^− 8^, *p* = 9.01e^− 9^, and *p* = 0.0005 for T12, T24, T48, and T96, respectively; Kruskal-Wallis), with trends becoming especially apparent after all quality control steps (Supplementary file 1 Figure S2). Therefore, to account for differences in library size among samples, the *rrarefy* function from the *vegan* package [73] was used to randomly subsample counts data to 5000 reads, as a majority of the observed richness was captured by 5000 reads (Supplementary file 1 Figure S3). For samples with fewer than 5000 total reads (70 samples with an average read depth of 3,404 reads), no random subsampling occurred, and all reads were used.

### Microbiome and statistical analyses

#### Alpha diversity and microbiome relative abundance

Observed Richness, Shannon Diversity, Inverse Simpson, and Faith’s Phylogenetic Diversity (PD) were all calculated at the genus level using the *estimate_richness* function in the *phyloseq* package. All alpha diversity measures were calculated using the rarefied phyloseq object described above. For brevity, Shannon Diversity is presented here at the genus level, although other metrics can be seen in Figure S5 in Supplementary file 1.

To quantitatively evaluate how coral fragments responded to each antibiotic treatment over time, a linear mixed-effect model was created using the *lme4* R package (v. 1.1.35.1) [74]. In this model, time (categorical variable), treatment (antibiotic plus dose), and their interaction were set as fixed effects, while tank and coral sample ID were set as nested, random effects (Supplementary file 1). Pairwise comparisons were made using the *emmeans* package (v. 1.10.0), with the Tukey-Kramer p-adjustment method [75].

Pruned, rarefied data were also used to calculate relative abundance measures for each antibiotic treatment group. After taxa counts were transformed to relative abundances, the top ten most abundant taxa across the samples were determined. Treatment replicates were then merged to calculate the mean relative abundance of the top ten most abundant taxa across all samples.

#### Beta diversity and dispersion

Beta diversity analyses were performed using pruned, unrarefied data that were robust centered log-ratio (rCLR) transformed data using the *microViz* package to account for data compositionality and sparsity [76, 77]. Transformed data were ordinated using a Principal Component Analysis (PCA). Differences in beta diversity between sample groups within time points were identified via a permutational analysis of variance (PERMANOVA) using *adonis2*. The *pairwise.adonis* package was used for pairwise comparisons, and p-values were adjusted according to the false discovery rate (fdr) formula [78]. Beta dispersion (as distance to centroid) was determined using Euclidean distances that were calculated from the rCLR-transformed dataset, thereby producing robust Aitchison distances. Statistically significant differences were determined using the *betadisper* and *permutest* functions in the *vegan* package with fdr p-value correction [73].

#### Differential abundance

Differential abundance analysis was performed using ANCOM-BC2 on pruned, unrarefied data to identify taxa whose relative abundances were significantly different [79]. For each treatment, all time points (T12 - T96) were combined and compared to all pretreatment (T0) samples. Repeated sampling was accounted for by including coral ID as a random effect in the differential abundance models.

#### Network analysis

Data subsets were created for each treatment using the original, unrarefied, unpruned phyloseq object. Time 0 samples were excluded from analysis, as corals at this time point had not yet been exposed to antibiotics. Before network construction, taxa that only appeared in one sample were removed. Microbial co-occurrence networks were then created using the *microeco* R package [80]. Networks were created using the SpiecEasi method with Meinhausen and Bühlmann (MB) neighborhood selection to calculate taxon co-occurrence at the ASV level [81]. For each antibiotic, samples from each dose and all time points (except for time 0) were combined, such that only one network per antibiotic was generated. Network centrality measures were calculated using functions within the *igraph* and *meconetcomp* packages [82, 83]. Networks were then filtered to only include the top three most relatively abundant ASVs and their interactions to understand how each treatment affected the co-occurrence relationships among them. These ASVs included *Campylobacterales* (ASV1), *Helicobacteraceae* Family (ASV3), *Phaeobacter* (ASV4). Full networks were visualized using Cytoscape [84] and can be viewed in Figure S8 in Supplementary file 1 or as interactive network plots on NDEx following the link provided in the availability of data and materials section below.

## Results

### Antibiotics reduce a dominant, unclassified *Campylobacterales* ASV

**Fig. 1:**
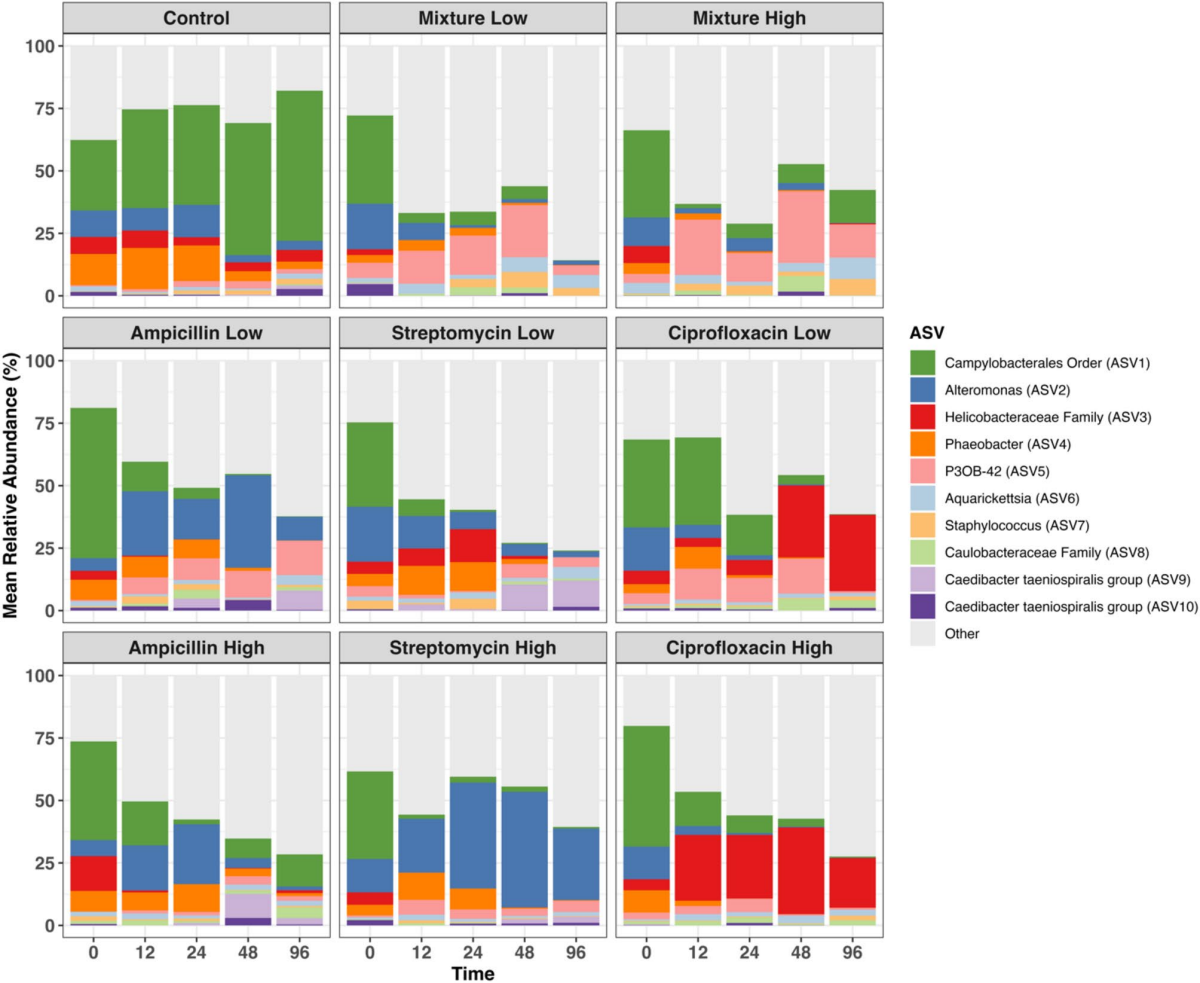
Mean relative abundance of the top ten most abundant ASVs across all antibiotic treatment groups. Each bar represents the average of a minimum of 19 replicates in the control treatment group, and a minimum of 9 replicates in each of the antibiotic treatment groups

Microbiomes of T0 (pre-treatment) corals, and those of all time points within the control treatment group, were dominated by a single bacterial taxon from the order *Campylobacterales* (ASV1) (Fig. 1). The mean relative abundance of this bacterium in control samples remained high over the course of the experiment, with mean relative abundance ranging from 28.25 ± 31.00% at T0 to 60.12 ± 29.18% at T96 (Fig. 1 and Supplementary file 2 Table S1). However, in all antibiotic treatment groups, regardless of dose, the mean relative abundance of ASV1 was markedly reduced (Fig. 1). Most antibiotic treatment groups showed a reduction of this taxon by 12 h (T12) after the initial antibiotic dose, and this reduction was maintained throughout the experiment.

Despite each antibiotic reducing the mean relative abundance of the uncharacterized *Campylobacterales* ASV, the bacterial taxa that then increased in relative abundance differed among antibiotic type and dose. For example, the ‘mixture low’ samples displayed higher relative abundances of a genus within the Myxococcaceae family, P30B-42, (13.15 ± 21.20% at T12) than control samples (1.07 ± 2.90% at T12) while a single *Alteromonas* taxon became more dominant in ‘ampicillin low’ and ‘streptomycin high’ samples after the initial antibiotic administration compared to control, mixture, and ciprofloxacin treated samples (Fig. 1). Although already found at relatively low abundance in both T0 (5.94 ± 10.42%) and control samples over time (5.04 ± 11.84%), a single ASV from the *Helicobacteraceae* family (ASV3) was almost entirely eliminated by T96 by all antibiotic treatments, except for in the ciprofloxacin treatment group in which the reverse pattern was identified (Fig. 1). By T96 for both the ‘ciprofloxacin low’ and ‘high’ treatment groups, this *Helicobacteraceae* taxon became the dominant taxon (30.52 ± 43.57% and 26.31 ± 26.59%, respectively) (Fig. 1). Detailed relative abundance patterns for individual corals are shown in Supplementary file 1 Figure S4.

### Time and antibiotic treatment differentially impact coral microbiome alpha diversity

**Fig. 2:**
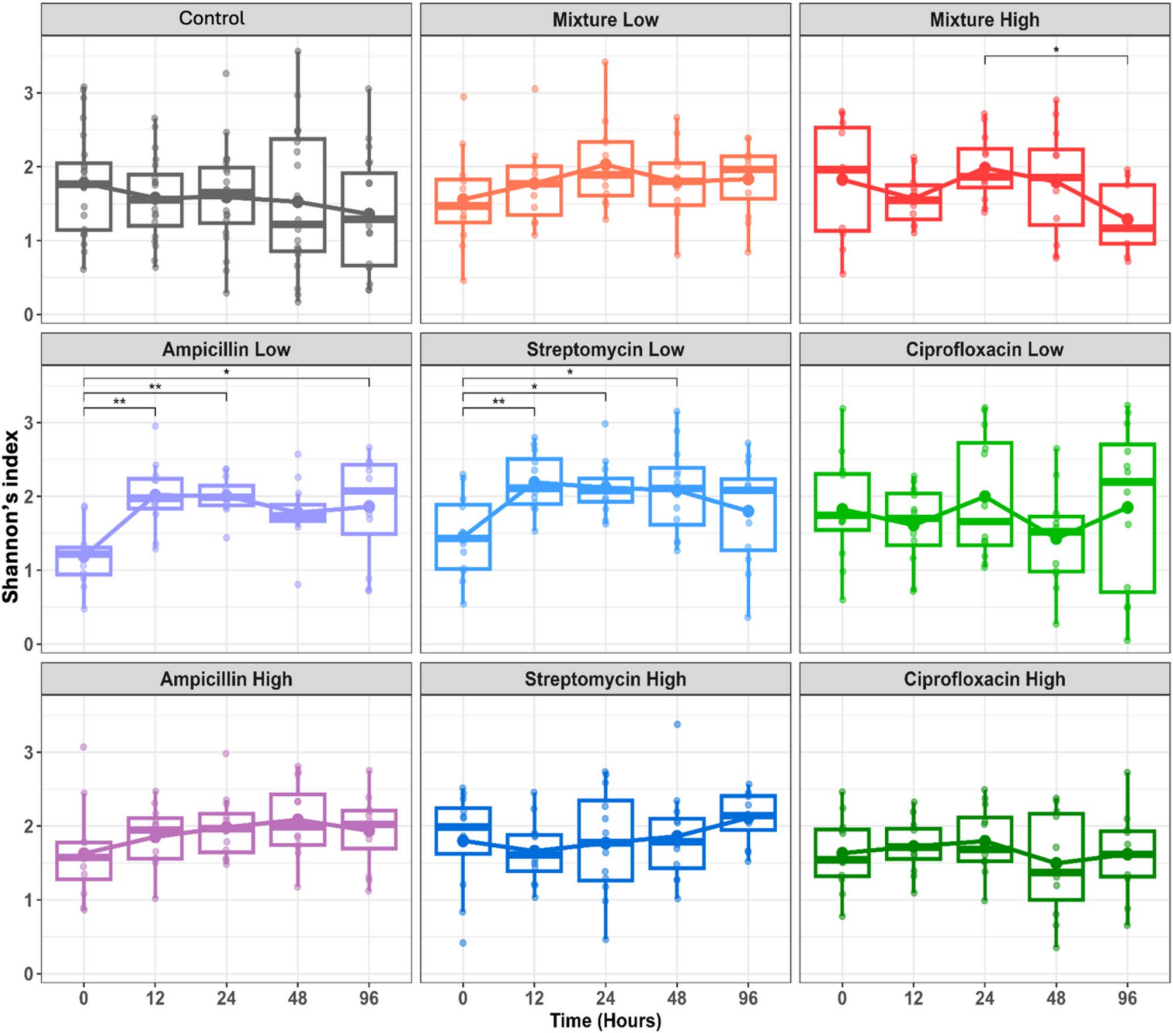
Shannon Diversity calculated at the genus level. Statistical significance was determined by a LMM in which treatment, time, and their interaction were set as fixed effects, while tank and unique coral ID were set as nested, random effects. Pairwise comparisons of estimated marginal means were calculated and the p-value was adjusted using the Tukey method. Significance codes are as follows: 0 ‘***’, 0.001 ‘**’, and 0.01 ‘0.01’

Statistical analyses of Shannon diversity revealed that time, and the interaction of time and treatment, significantly affected the combined richness and evenness (Shannon diversity) (LMEM, *p* = 0.002; Supplementary file 2 Table S2). Upon further pairwise analysis, although time was a significant driver of differences in Shannon diversity, no significant differences were observed in the control treatment group over time (Fig. 2, Supplementary file 2 Table S3). Similarly, no significant differences in Shannon diversity between time points in ciprofloxacin-treated samples were identified for either dose (Fig. 2). For ampicillin and streptomycin, changes in alpha diversity appeared to be largely dose-dependent, as significant increases in Shannon diversity were observed only in the low doses of each (Fig. 2). In ‘ampicillin low’ samples, at time points T12, T24, and T96, Shannon diversity increased significantly compared to T0 (Fig. 2, Pairwise EMM, *p* = 0.001, *p* = 0.002, and *p* = 0.02, respectively; Supplementary file 2 Table S3). ‘Streptomycin low’ samples similarly displayed increased alpha diversity over time compared to the pre-treatment time point with significant differences at T12, T24, and T48 (Fig. 2, Pairwise EMM, *p* = 0.006, *p* = 0.018, *p* = 0.03, respectively; Supplementary file 2 Table S3). Unlike ampicillin and streptomycin, where significant increases in alpha diversity were observed in the low dose, one significant comparison was found in the high-dose mixture treatment group between T24 and T96 in which diversity was reduced in the later time point (Fig. 2, Pairwise EMM, *p* = 0.026, Supplementary file 2 Table S3). Although not statistically significant, ‘mixture high’ was the only treatment group that displayed decreased diversity at T96 compared to T0 (Fig. 2).

### Antibiotic treatment results in distinct shifts in coral microbiome beta diversity

PERMANOVA identified time, treatment, and the interaction between time and treatment as drivers of differences in beta diversity (Supplementary file 2 Table S4). Time also significantly affected beta diversity in control samples (Supplementary file 2 Table S4). Therefore, comparisons of community dissimilarity were made within time points, to identify significant differences between antibiotic doses compared to untreated samples subjected to the same tank residence time.

**Fig. 3:**
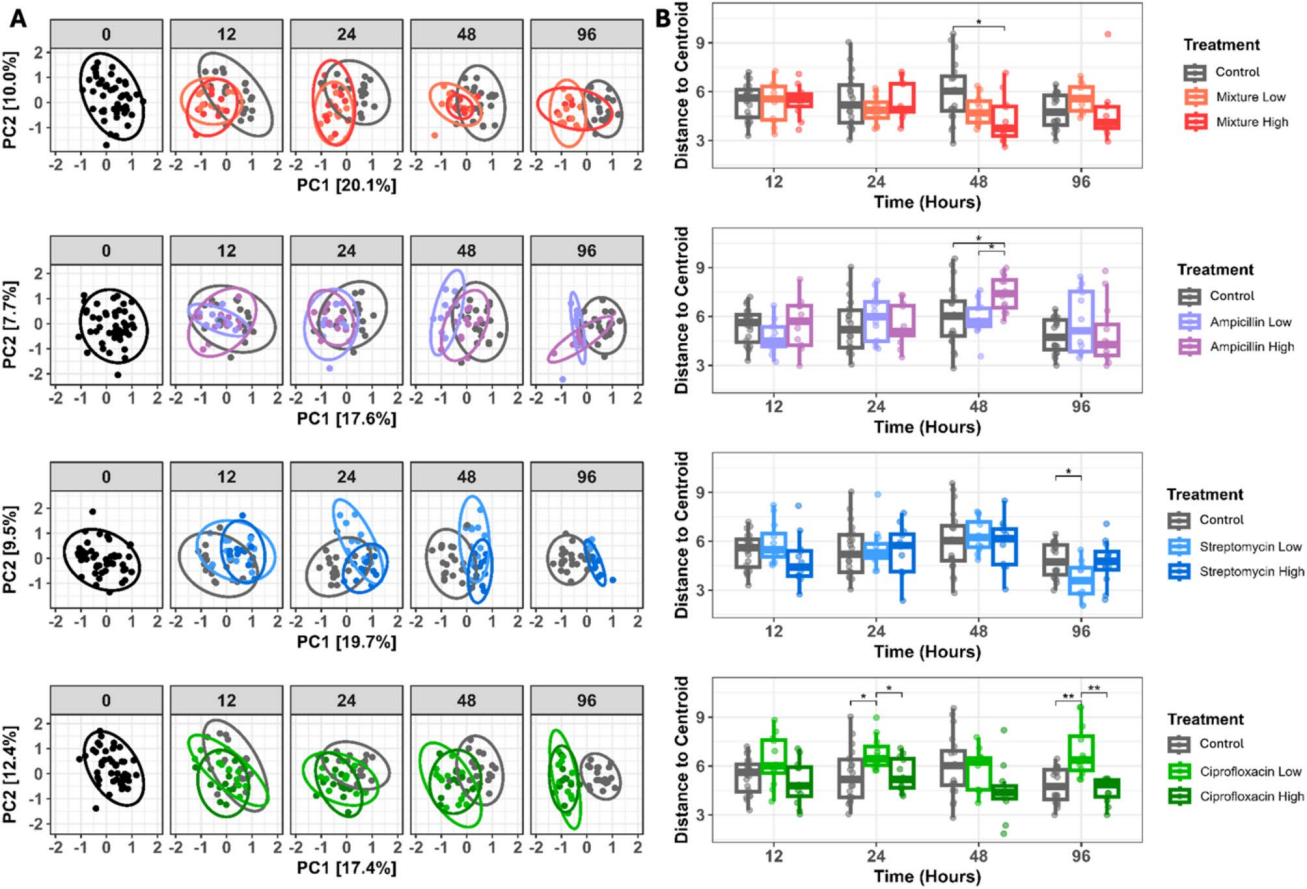
Principal components analysis ordination of beta diversity based on Euclidean distances of robust centered log-ratio transformed data (**A**). Control, high, and low doses of each antibiotic were compared within each time point. Ellipses display 95% confidence intervals. Black dots and ellipses at time 0 represent all samples prior to the treatment. Beta dispersion, as distance-to-centroid, by antibiotic treatment group within a time point (**B**). Significance levels reflect fdr-adjusted p-values. Significance codes are as follows: 0 ‘***’, 0.001 ‘**’, and 0.01 ‘*’

At each time point, aside from T0, significant differences in beta diversity were observed between both doses of the antibiotic mixture and streptomycin relative to control samples (Fig. 3). Dose-dependent community differences were detected at T96 between ‘mixture low’ and ‘mixture high’ (*p* = 0.03; Fig. 3; Supplementary file 2 Table S5), and at T24, T48, and T96 between ‘streptomycin low’ and ‘streptomycin high’ (*p* = 0.001; Fig. 3; Supplementary file 2 Table S5). For ampicillin-treated samples, significant differences in beta diversity were detected beginning at T24 where ‘ampicillin low’ and ‘ampicillin high’ community profiles were distinct from control samples (*p* = 0.002), yet not significantly different from one another (Fig. 3, Supplementary file 2 Table S5). At times 48 and 96, however, ‘ampicillin low’, ‘ampicillin high’, and control groups displayed significantly different microbiome community structures– indicating both treatment and dose-dependent responses at these later time points (*p* = 0.001 at T48, *p* = 0.002 at T96, Fig. 3, Supplementary file 2 Table S5). The community composition of the ‘ciprofloxacin high’ samples significantly differed from that of the control samples at T12 (*p* = 0.012, Fig. 3), yet not for the low dose. At all subsequent time points, both ‘ciprofloxacin high’ and ‘ciprofloxacin low’ samples exhibited distinct community clustering from untreated samples (*p* = 0.002, Fig. 3), although no dose-dependent differences were observed.

All antibiotics also displayed significant differences in within-group variation, or beta dispersion, in at least one time point. The direction of differences in dispersion, however, depended on the antibiotic as well as dose. At time T48, ‘mixture high’ samples displayed significantly reduced dispersion compared to control samples (*p* = 0.027, Fig. 3), while ‘ampicillin high’ displayed significantly increased dispersion compared to control and ‘ampicillin low’ samples (*p* = 0.03 and *p* = 0.003, respectively, Fig. 3). ‘Streptomycin low’ samples had significantly lower beta dispersion compared to control samples at T96 (*p* = 0.02, Fig. 3, Supplementary file 2 Table S6), while ‘streptomycin high’ was not distinct from the control group. Low-dose ciprofloxacin samples displayed increased dispersion compared to control and high-dose samples at both T24 (*p* = 0.018 and 0.03, respectively) and T96 (*p* = 0.0015, Fig. 3).

### Patterns in coral bacterial differential abundance are dependent upon antibiotic type and dose

**Fig. 4:**
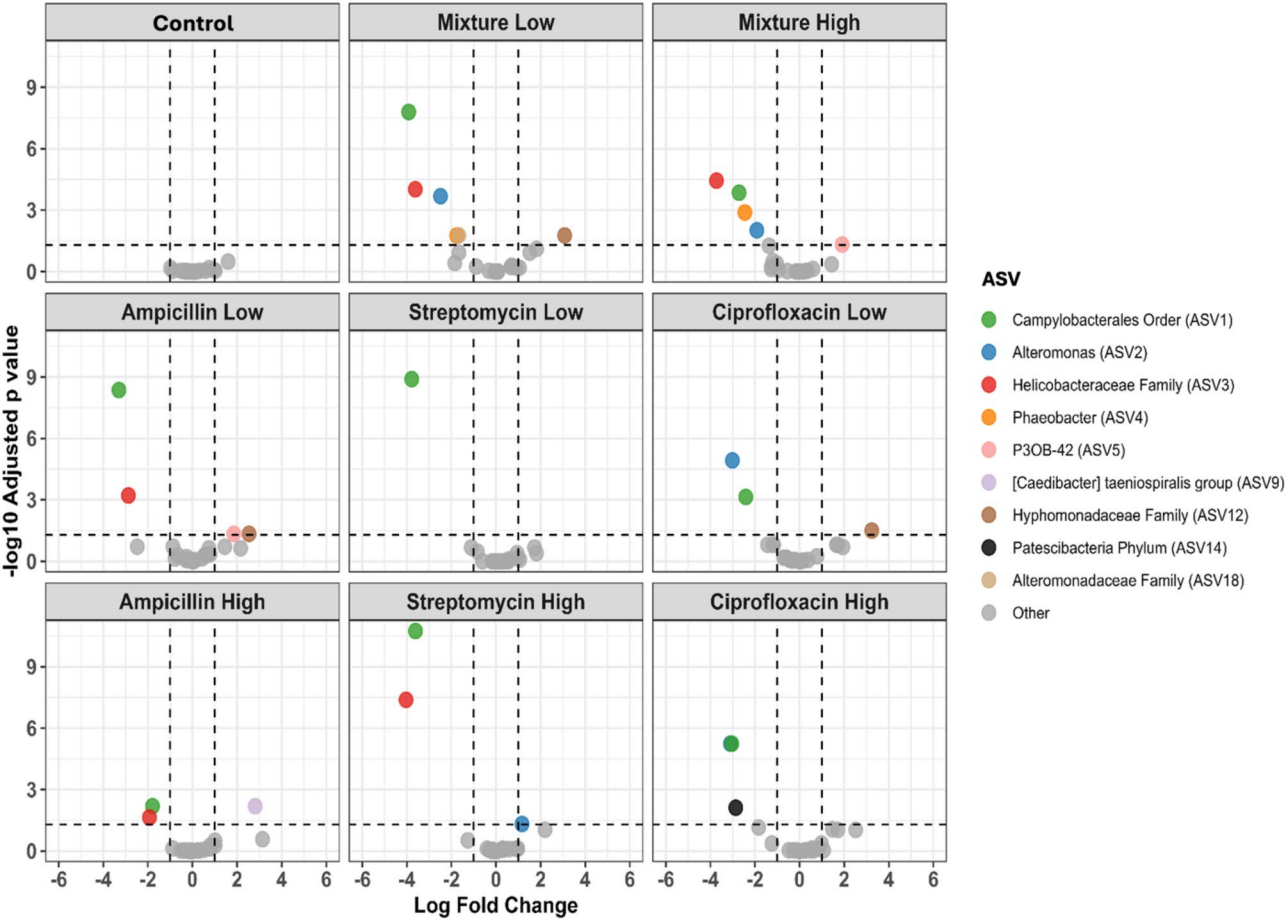
Volcano plot depicting differentially abundant ASVs by treatment, as determined by ANCOM-BC2. Each treatment group was compared against all pretreatment samples (time 0). Points below the horizontal dotted line were not significantly differentially abundant. Each panel contains all samples from times 12 through 96 within that antibiotic treatment group

No taxa were differentially abundant across time in the control samples (Fig. 4, Supplementary file 1 Figure S6). However, variable patterns in differential abundance were seen based on antibiotic type and dose. Every antibiotic treatment, at all doses, resulted in a significant reduction of the unclassified *Campylobacterales* ASV1 (Fig. 4), with the largest negative log-fold change occurring in ‘mixture low’ with a log-fold change of -3.9 (Supplemental Table 7). At least one dose within each antibiotic group, with the exception of ciprofloxacin, also resulted in the reduction of ASV3 from the *Helicobacteraceae* family (also within the *Campylobacterales* order) (Fig. 4). ‘Mixture high’ and ‘mixture low’ and ‘ciprofloxacin high’ and ‘ciprofloxacin low’ also all displayed reduced abundance of *Alteromonas* (ASV2), while ‘streptomycin high’ was the only group in which this taxon increased in relative abundance.

Overall, samples treated with the antibiotic mixture displayed the largest number of taxa that were significantly reduced, with five and four taxa reduced in ‘mixture low’ and ‘mixture high’ groups, respectively (Fig. 4). A taxon from the genus *Phaeobacter* (ASV4) (formerly *Nautella*) was reduced in both mixture doses, and a taxon from the *Altermonadaceae* family (ASV18) decreased in ‘mixture low’ (Fig. 4). Apart from the *Alteromonas* taxon (ASV2) that had significantly higher relative abundance in ‘streptomycin high’ samples, only three other taxa were significantly increased by antibiotic treatments. A taxon from the family *Hyphomonadaceae* (ASV12) increased significantly in ‘mixture low’, ‘ampicillin low’, and ‘ciprofloxacin low’ samples relative to pre-treatment samples. ‘Mixture high’ and ‘ampicillin low’ both displayed elevated abundances of a taxon from the *Caedibacter taeniospiralis* group (ASV9) (Fig. 4). Differential abundance patterns for each treatment over time can be seen in Supplemental Fig. 6.

### Minor taxa primarily drive network structure and function

**Fig. 5:**
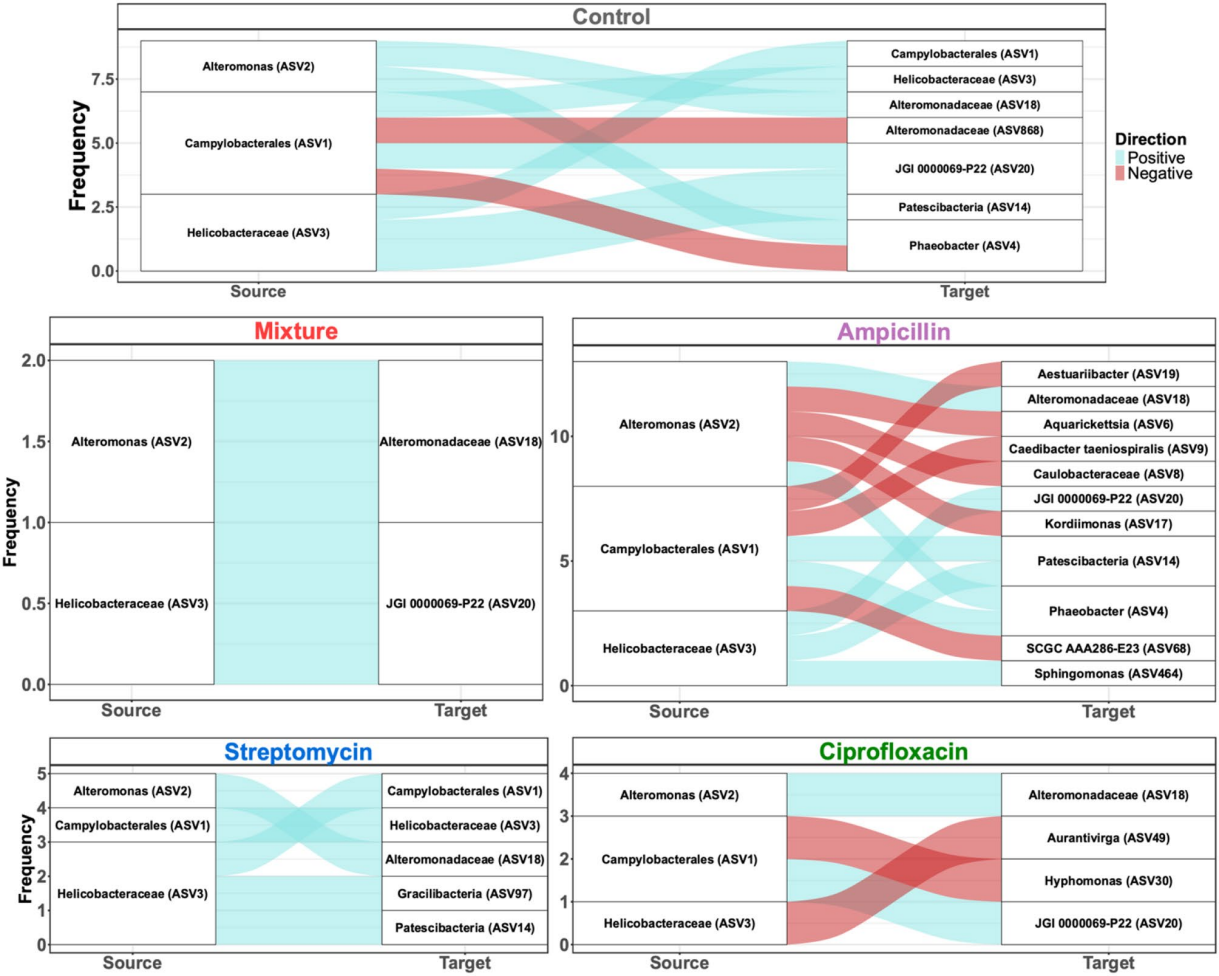
Alluvial plot displaying positive and negative microbial co-occurrence patterns among the top three most relatively abundant ASVs (*Campylobacterales* order ASV1, *Alteromonas* ASV2, and *Helicobacteraceae* family ASV3). Co-occurrence patterns were determined using SpiecEasi

Each network was subset to only include the top three most abundant ASVs (*Campylobacterales* order ASV1, *Alteromonas* ASV2, and *Helicobacteraceae* family ASV3) to observe how their relationships changed across networks. Several positive co-occurrence relationships observed in the control network were also seen across the treatment networks. The co-occurrence between the *Alteromonas* ASV2 and an unclassified ASV18 from the *Alteromonadaceae* family was seen in each of the networks (Fig. 5), and was, in fact, the only relationship conserved in all networks. Interestingly, although this interaction was present in each of the networks, its structural and/or functional importance does not appear consistent among the networks, as the relationship between ASV18 and ASV2 forms a distinct cluster away from the main network in the mixture network, whereas the co-occurrence relationship is more central in all other networks (Supplementary file 1 Figure S8).

Despite being the most abundant taxon in the control samples, *Campylobacterales* ASV1 was not identified as a microbiome network hub node in any of the five networks according to centrality metrics such as degree, closeness centrality, or eigenvector centrality (Supplemental Table 10). This taxon had eigenvector centrality scores of 7.99e-08, 4.77e-03, 2.37e-04, and 2.7e-04 (scores range from 0 to 1, with more influential taxa having scores closer to 1) for control, ampicillin, streptomycin, and ciprofloxacin networks, respectively, and did not have any significant connections in the mixture network. Instead, those taxa with eigenvector centrality scores of 0.75 or higher were considered hub nodes.

Hub nodes were identified as *Chryseobacterium* (ASV664) and *Winogradskyella* (ASV25) in the control network, *Caenarcaniphilales* Order ASV406 and *Woesia* (ASV232) in the mixture network, *Aquibacter* (ASV37), ASV654 from the *Stappiaceae* family, and *Labrenzia alexandrii* (ASV367) in the ampicillin network, *Pseudoaminobacter* (ASV86) and *Aestuariibacter* (ASV1057) in the streptomycin network, and *Tropicibacter* (ASV329) and ASV411 from the *Cryomorphaceae* family in the ciprofloxacin network (Supplementary file 1 Figure S9). Degree was also measured to identify highly connected taxa. The taxa with the most connections in the control, mixture, ampicillin, streptomycin, and ciprofloxacin networks were *Blastopirellula* (ASV503) and *MBIC10086* (ASV2773) (degree = 11), *Dadabacterales* order ASV485 (degree = 8), *Muricauda* ASV21 (degree = 11), *Pseudoalteromonas* (ASV555) (degree = 14), *Rhodobacteraceae* family (ASV263) (degree = 16), respectively.

### Water and coral microbiome compositions remain distinct from one another before and after antibiotic treatment

**Fig. 6:**
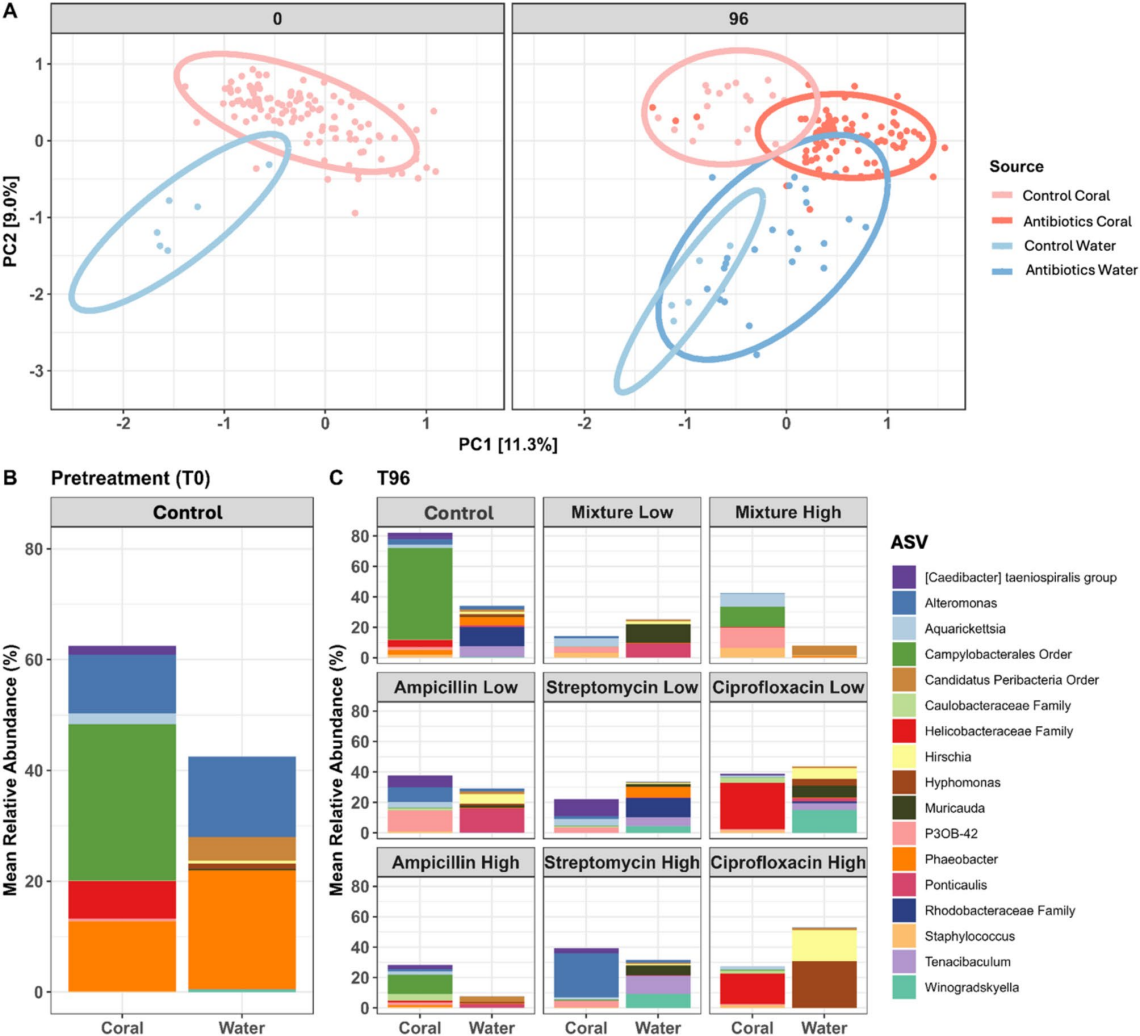
Combined PCA ordination (**A**) and mean relative abundance plot depicting the top ten most abundant taxa in coral and top ten most abundant taxa in water samples at time 0 (**B**) and time 96 (**C**). Beta diversity was calculated based on Euclidean distances of robust centered log-ration transformed data. Ellipses represent 95% confidence intervals. In panel B, each bar represents the average of six samples for water, and 119 samples for coral. In panel C, each water sample bar represents the average of three samples for experimental treatments and five samples for control. Each coral sample bar represents the average of 19 samples for control, 12 samples for ‘ampicillin low’, ‘ampicillin high’, ‘ciprofloxacin low’, ‘mixture low’, and ‘streptomycin high’, 11 samples for ‘ciprofloxacin high’ and ‘streptomycin low’, and 9 samples for ‘mixture high’

Prior to antibiotic treatment, coral and water samples displayed distinct microbiome structures (Fig. 6A and B). After the 96-hour antibiotic exposure experiment, the coral and water samples from the control samples maintained this separation (Fig. 6). Furthermore, antibiotic exposure appeared to shift both water and coral microbial communities away from their respective untreated control groups (Fig. 6A). Interestingly, antibiotic administration appeared to induce a shift in which bacterial community diversity between coral and water samples became more similar, although each group remained significantly dissimilar from one another (Fig. 6A, Supplementary file 2 Table S8). This convergence, however, appears to be driven by the ampicillin groups, as the coral and water were more similar in this treatment than in other antibiotic groups (Supplemental Fig. 7), or due to the residence time of the corals in the aquaria at the final time point. Additionally, we found that the antibiotic-induced loss of the dominant *Campylobacterales* ASV in corals was not mirrored in the water samples, nor was that taxon found to be established in the water column following depletion from the coral host (Fig. 6C). In fact, the *Campylobacterales* ASV was present at very low abundance and prevalence in all water samples. This ASV was found in 83% of coral samples and accounted for 23.6% of the total reads, while it was found in 48.6% of water samples and only contributed to 0.04% of the total reads. Genera such as *Alteromonas* and *Phaeobacter* that were identified in coral samples, were identified in relatively high abundance in water samples (Fig. 6). Yet, interestingly, these taxa were largely eliminated from the water column by each antibiotic treatment, whereas the reduction of these taxa was more varied across treatment groups in the coral samples (Figs. 1 and 6).

## Discussion

In this study, we found that a highly relatively abundant bacterium in the microbiome of a disease resistant *Acropora cervicornis* genotype is highly susceptible to antibiotics. As a result of the widespread reduction in the *Campylobacterales* ASV, alpha diversity increased in many cases, and several bacterial ASVs increased in relative abundance– suggesting antibiotic-induced dysbiosis and a potential supplementation of opportunist bacteria. Antibiotic treatment significantly altered bacterial community structure and diversity by both antibiotic type and antibiotic dose, and network analysis revealed that minor taxa are likely playing significant roles in the coral microbiome structure and function.

### *Campylobacterales* in disease-resistant *Acropora cervicornis*

Genera within the order *Campylobacterales*, namely *Campylobacter* and *Arcobacter*, have been associated with a range of diseases in various coral species [34, 85–90], although none have been implicated as primary pathogens. Furthermore, *Campylobacterales* metagenome-assembled genomes (MAGs) most closely related to the genus *Arcobacter* were enriched in diseased coral tissue compared to apparently healthy tissue samples [87]. However, since 2019, an uncharacterized ASV belonging to the order *Campylobacterales* dominated the microbiomes of some specific genotypes of *A. cervicornis*, particularly in the same genotype studied here; however, there is no evidence suggesting that these corals are diseased [91, 93]. In fact, this genotype is one of the few *A. cervicornis* genotypes found to be disease-resistant both ex situ and in situ [90–92, 93 E. Bartels, *pers. comm.*]. Interestingly, despite the high relative abundance of *Campylobacterales*, this dominance is thought to represent a shift in microbiome composition over time. In samples collected from 2015, corals of this same genotype displayed far more even and diverse microbiome with very low abundance of this *Campylobacterales* ASV [95].

Since 2015, there have been no reports that this genotype has displayed any changes in its disease resistant phenotype. Therefore, given this shift toward a *Campylobacterales*-dominated microbiome, there may exist at least two microbiome states that are capable of supporting disease resistance in *A. cervicornis*, yet the implications of reduced microbiome evenness remain unclear and further disease experiments are needed. It is possible that the shift toward single-taxon dominance may ultimately be detrimental to the system, as observed in disease-susceptible *A. cervicornis* genotypes dominated by the intracellular bacterial parasite *Ca*. *Aquarickettsia rohweri* [95]. More likely, the association of this *Campylobacterales* taxon may be indicative of a newly established, potentially beneficial, symbiotic relationship in response to chronic nutrient enrichment on reefs, as many taxa within the *Campylobacterales* order are important contributors to sulfur and nitrogen cycling [96–98]. Some members reduce nitrate to ammonium, which is potentially important on reefs as nitrate enrichment alone is known to exacerbate thermal bleaching outcomes and hinder coral growth rates, while slight ammonium enrichment, coupled with natural sources of phosphorous, may be beneficial to coral growth [91, 96, 99, 100]. In a recent study, the microbiome of the same *A. cervicornis* genotype used in the present study was stable in response to acute nutrient enrichment, including the maintenance of *Campylobacterales* dominance throughout treatment [91]. Therefore, it was hypothesized that the microbiome structure of this disease-resistant genotype may provide some tolerance to environmental stressors [91]. Interestingly, despite the significant loss of this dominant ASV following the antibiotic challenge, there were no immediate, visible signs of host health decline (i.e., no loss of pigment or tissue sloughing), no striking changes in co-occurrence relationships of this taxon among networks, nor were there drastic shifts in the number of taxa co-occurring with this ASV. This may indicate that the *Campylobacterales* ASV1 is not a species generalist in the microbiome, as it only interacts with a select few other taxa in the networks [101]. Given its rapid loss, yet concomitant apparent host health stability, we hypothesize that the *Campylobacterales* taxon itself is not required to maintain disease resistance and is rather a nonobligatory association.

### Reduction in a *Campylobacterales *ASV potentially supports an increase of other taxa

In this study, we show that the *Campylobacterales* ASV1 was highly susceptible to all antibiotic treatments (Fig. 4) and that taxa associated with coral stress response increase in relative abundance to presumably inhabit the niche space that the *Campylobacterales* occupied. The second-most reduced ASV in this study was from the *Helicobacteraceae* family, which is also within the *Campylobacterales* order, and which was reduced in all treatment groups except for ciprofloxacin and ‘streptomycin low’ (Figs. 1 and 4). Given the constraints of relative abundance analyses and compositional data, it is difficult to discern whether there are true increases in taxa following a reduction in a more dominant ASV, or if they simply appear to increase. Therefore, without measuring total bacterial abundance, the conclusions drawn from changes in taxa relative abundance limit our understanding of the true implications of antibiotics on the microbiome. To definitively track these changes in bacterial abundance, methods such as quantitative or digital PCR are needed, although the methods and costs to do so also limit their approachability for such a large, nested study.

Among the taxa that were positively enriched in response to antibiotic treatment, P3OB-42 (ASV5; *Myxococcales*) and *Caedibacter taeniospiralis* (ASV9) are of particular interest. The taxon P3OB-42 is hypothesized to play a role in pathogen regulation in *A. cervicornis*, as higher relative abundances of this taxon were associated with corals that were exposed to disease, yet remained visually unaffected [102]. In a plant agricultural system, *Myxococcales* spp. were also shown to inhibit phytopathogen infection [103]. Given the putative commensal nature of *Myxococcales*, the increase in this taxon may result from antibiotic-induced disruption to the coral surface mucus layer (SML), thereby exposing the coral to potential invasion as the SML normally serves as a first line of defense via niche occlusion and antimicrobial properties [18, 104]. Interestingly, species within the P3OB-42 genus are also known antibiotic degraders– specifically sulfonamides and beta-lactam antibiotics [105, 106]– which may explain the significant enrichment of this taxon in response to a low dose of ampicillin and high dose of the antibiotic mixture (Fig. 4).

The other notable bacterium that increased in relative abundance following antibiotic treatment was from the *Caedibacter taeniospiralis* group (closely related to the genus *Cysteiniphilum*: BLASTn 100% sequence identity), which is a known obligate intracellular symbiont of the paramecium *Paramecium tetraurelia*, capable of conferring a fitness advantage to the host by producing refractile bodies that kill other paramecia [107, 108]. Although this paramecium has not been documented in corals– healthy or diseased– ciliated protozoans are often associated with disease incidence [89, 109, 110]. Recently, the *Caedibacter taeniospiralis group* was also identified at higher abundances in diseased corals exposed to both white band disease type I and the coral pathogen, *Serratia marcescens* [111]. In the present study, and in Young et al., 2023, the *Caedibacter taeniospiralis group* had lower abundance in controls. Similarly, the closely related taxon, *Cysteiniphilum litorale* was found in significantly higher relative abundance in WBD-afflicted *A. cervicornis* (77.5% ± 5.1% SE) compared to healthy corals (2.9% ± 1.2% SE) [112]. Using machine learning and transmission experiments, this ASV was further identified as a potential WBD pathogen [112], thereby indicating that the increase of this taxon is likely due to burgeoning opportunistic establishment.

### Dose-dependent differences in bacterial responses

Our results suggest that care must be taken to identify the most appropriate antibiotic dose to treat coral disease, as we found that different antibiotics result in dramatically different microbial community structures compared to control samples. Further, we found that increased dose does not simply magnify the effect of change but rather can result in strikingly different community compositions– especially after prolonged exposure (Fig. 3). This dose-dependent effect was demonstrated by an *Alteromonas* ASV, which was reduced by the low dose of streptomycin (although not significantly), yet paradoxically proliferated in the high dose (Figs. 1 and 4). A BLASTn search revealed that this ASV shared 100% sequence identity with *Alteromonas macleodii* (e-value = 4e^− 127^), and strains within this species are known to be either fully resistant to antibiotics including streptomycin or only slightly sensitive to others such as ampicillin [113]. Given this resistance, it is likely that, in conjunction with the effects of antibiotic photodegradation, the low streptomycin treatment may have been administered at an effective dose. In the high dose, however, resistant strains of this taxon were possibly able to overcome the high concentration of antibiotics and then actively outcompete other bacteria [114]. It must be noted, however, that there is great diversity at the strain level within *A. macleodii*; therefore, without additional genomic information, the mechanisms of this shift remain largely unknown [115].

### Implications of antibiotic use

Despite their persistence and accumulation in the environment, antibiotics in aquatic systems are subject to various degradation mechanisms that further affect concentration. Photodegradation, hydrolysis, microbial degradation, and changes in pH and temperature, all contribute to reducing an antibiotic’s half-life [116–118]. In targeted therapeutic applications, these processes pose additional challenges for proper dosing which, as seen in this study, can drastically affect microbiome diversity and composition. Many of these processes, however, do not eliminate antibiotics entirely. Instead, these antibiotics are often reduced to subinhibitory concentrations, which may favor a shift toward microbial antibiotic tolerance and persistence in the system, thereby further complicating disease control [119].

Many coral diseases either have unknown etiological agents or are thought to be polymicrobial [34, 43, 85]. For this reason, broad-spectrum antibiotics are often employed to target a wide range of bacteria, but their use may have unintended ramifications. Given the variable range of inhibitory concentrations that antibiotics have on specific bacteria, it is difficult to develop a directed therapy that evenly targets each taxon of interest. Perhaps an antibiotic effectively targets one member of the polymicrobial consortia, which in turn provides the opportunity for an antibiotic-degrading bacteria to flourish, and potentially shield the other disease-associated taxa. In a recent study, researchers reported that normally commensal, beta-lactam degrading bacteria in the mouse gut may inadvertently protect a normally antibiotic-sensitive pathogen from the effects of ampicillin through commensal-mediated pathogen shielding [120]. Although the aforementioned study was conducted in a mouse model, similar mechanisms may be present in other systems. Given that antibiotic treatment does not always result in permanent disease cessation, this may indicate that one or more of the suspected pathogens may be initially susceptible but ultimately receive a level of protection due to antibiotic-degrading bacteria, or acquire resistance through horizontal gene transfer, thereby allowing the pathogen(s) to proliferate further.

Disturbances such as antibiotic treatment can destabilize a microbiome not only due to the direct bactericidal effects, but also in ways that transform microbe-microbe interactions. Increased positive co-occurrence patterns may present increased opportunities for positive feedback loops and unchecked proliferation in the microbiome, which are hypothesized to negatively affect microbiome stability, whereas competitive relationships are thought to assist in stability [11, 121]. Therefore, in addition to understanding how antibiotics affect the microbiome composition of target and off-target species, intervention strategies must also understand the effects these treatments have on microbial interactions as a whole.

## Conclusions

In this study, we found that following antibiotic perturbation, the abundance of a dominant, unclassified *Campylobacterales* taxon was significantly reduced by each antibiotic and dose. Despite varying implications of *Campylobacterales* in coral disease, the taxon described here does not appear to be associated with negative health effects in this coral genotype, although its capacity for commensalism and the implications of its loss remains unknown. Given the ecologically threatened state of many corals, antibiotics may provide a short-term approach to slow disease progression, yet dose range finding, off-target effects, and understanding how microbiome manipulation may affect a host’s long-term ability to combat future disturbances must be taken into consideration when assessing the risks and rewards of this approach. We also emphasize that although antibiotics have shown promise in disease mitigation, deliberately adding antibiotics to the environment should not serve as a permanent solution, and ethical considerations must be taken to understand the global, long-term implications of intervention strategies.

## Electronic supplementary material

Below is the link to the electronic supplementary material.

Supplementary Material 1

Supplementary Material 2

## Data Availability

Scriptsusedforbioinformaticandstatisticalanalysescanbefoundat: https://github.com/pattonsunni/RoL_Antibiotics_G7. The 16S rRNA dataset supporting the conclusions of this article is available in the NCBI Sequence Read Archive (SRA) repository under the BioProject accession number PRJNA1165811. Interactive networks can be accessed by the following link https://www.ndexbio.org/#/networkset/bec779a5-7d0f-11ef-ad6c-005056ae3c32?accesskey=ee1db6b84a489b391e5fbcbc8cdf1ac71becd0eabcc5b84ee52b30822da26a74.
