# Supplementary material for: Antibiotic type and dose variably affect microbiomes of a disease-resistant Acropora cervicornis genotype
Source: Environ Microbiome. 2025 May 2;20:46. doi: 10.1186/s40793-025-00709-2 (PMC12049008; doi:10.1186/s40793-025-00709-2)
Supplement: Supplementary file 1 — Supplementary Material 1 [file 40793_2025_709_MOESM1_ESM.docx]

Supplementary material for

**“Antibiotic type and dose variably affect microbiomes of a disease-resistant *Acropora cervicornis* genotype”**

Sunni Patton, Denise P. Silva, Eddie Fuques, Grace Klinges, Erinn M. Muller, Rebecca L. Vega Thurber

**
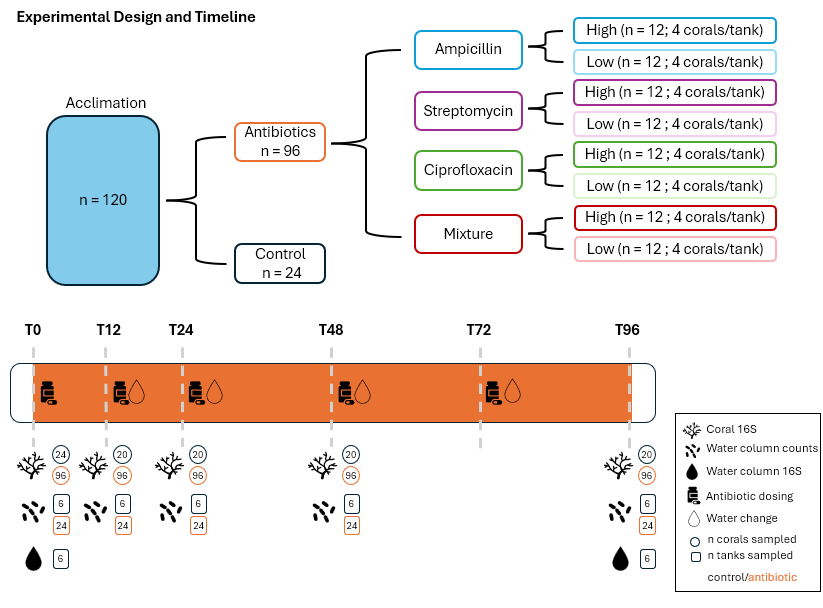
**

**Fig. S1** Experimental design, treatment timeline, and sample numbers for the antibiotic experiment.

**
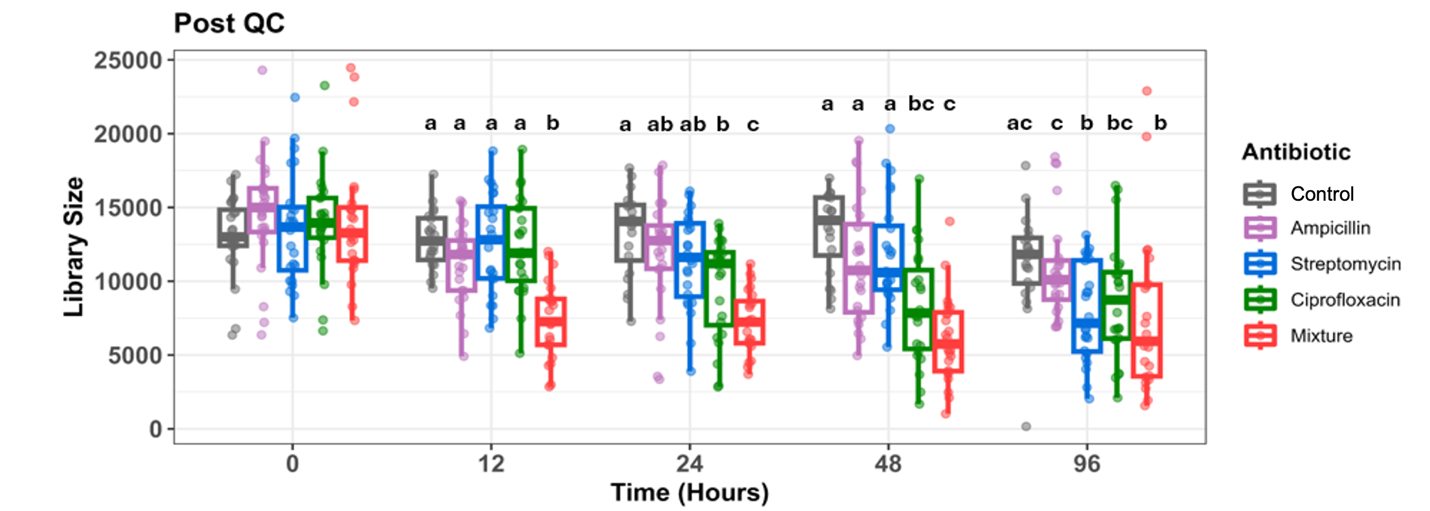
**

**Fig. S2** Boxplot displaying library size for all samples by antibiotic type. The top panel shows library sizes prior to quality control, while the bottom panel displays library sizes after all quality control steps. The Kruskal-Wallis test was used to determine significant differences between antibiotic treatments within each time point. The Dunn test with Benjamini-Hochberg correction was performed following a significant Kruskal-Wallis p-value. If boxplots share a letter, they are not significantly different from one another.


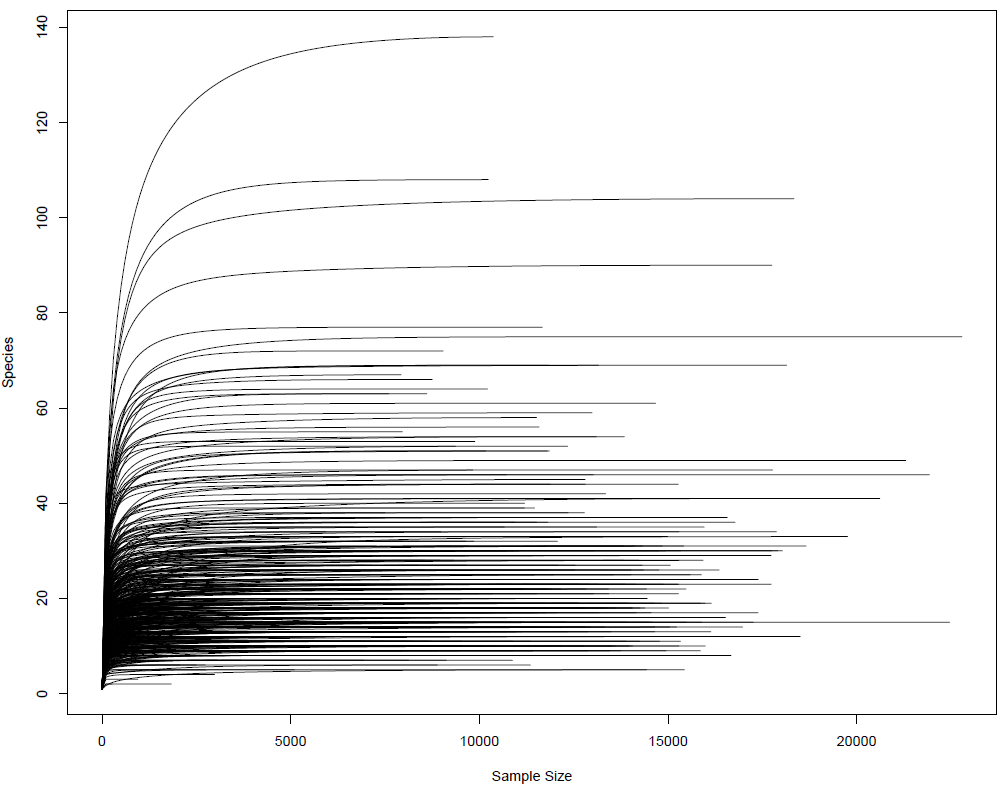


**Fig. S3** Rarefaction curve displaying the sampling depth at which the majority of the microbial diversity (in terms of observed richness) was captured.


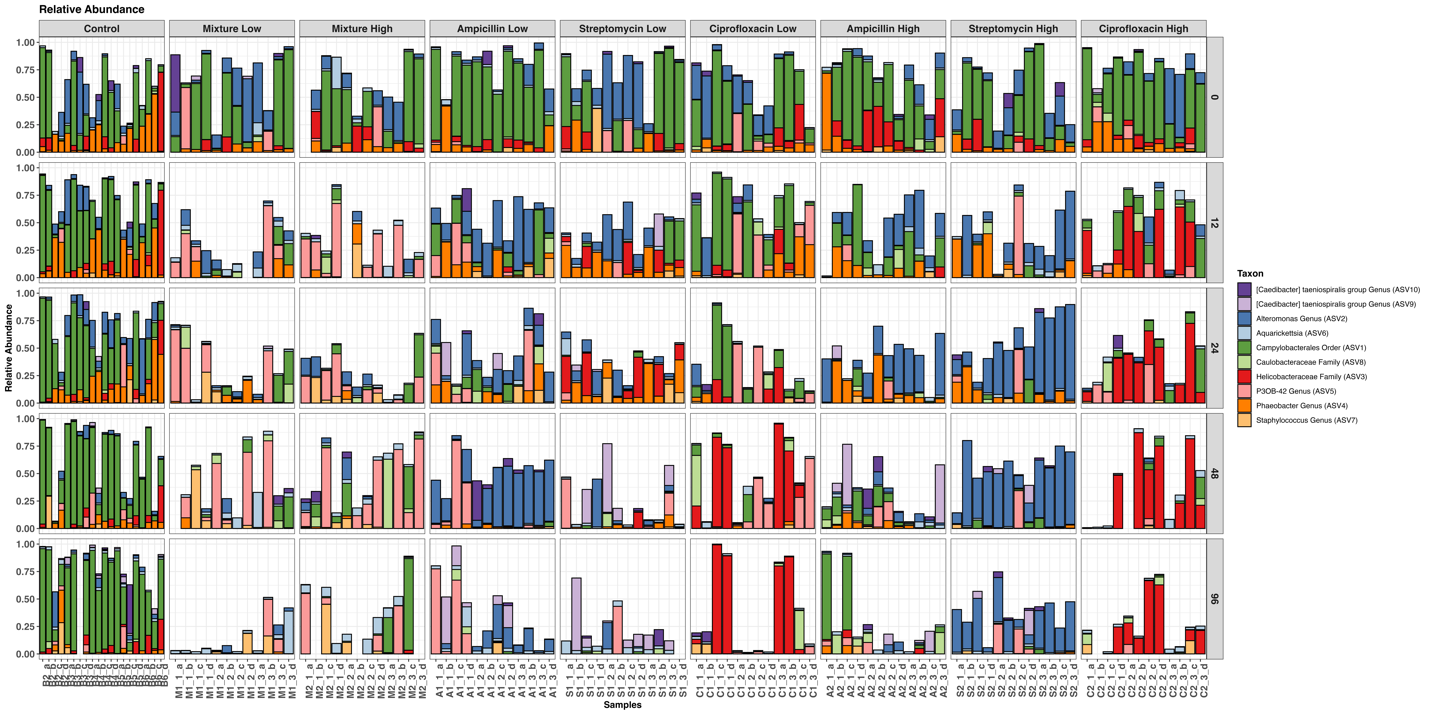


**Fig. S4** Mean relative abundance of the top ten most abundant taxa in individual coral fragments over the course of the experiment.


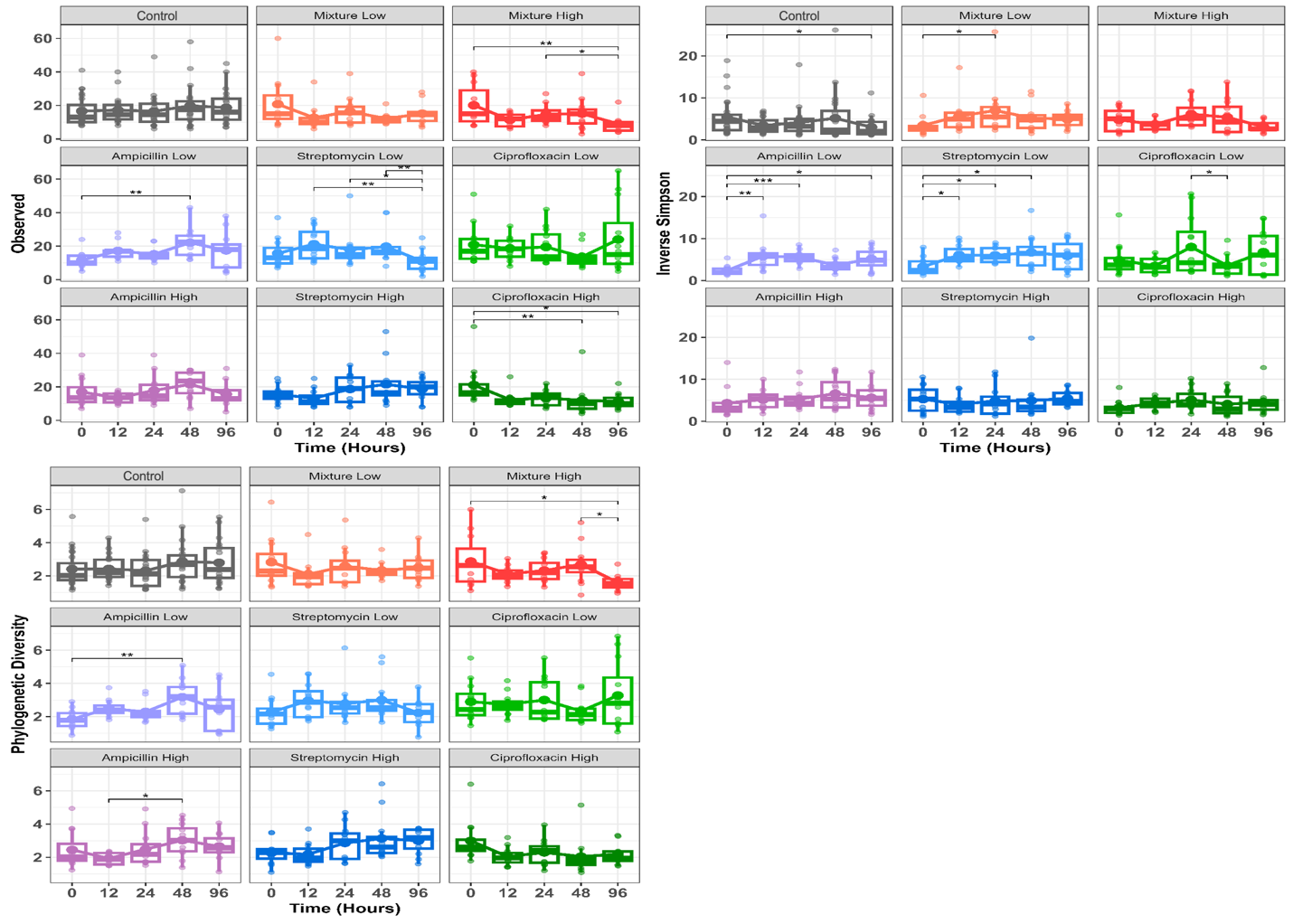


**Fig. S5** Observed richness, Inverse Simpson, and phylogenetic diversity calculated at the genus level. Statistical significance was determined by a linear mixed effects model (LMM) in which treatment, time, and their interaction were set as fixed effects, and tank and unique coral ID were set as nested, random effects. Pairwise comparisons of estimated marginal means (EMM) were calculated and the p-value was adjusted using the Tukey method. Significance codes are as follows: 0 ‘***’, 0.001 ‘**’, 0.01 ‘*’.


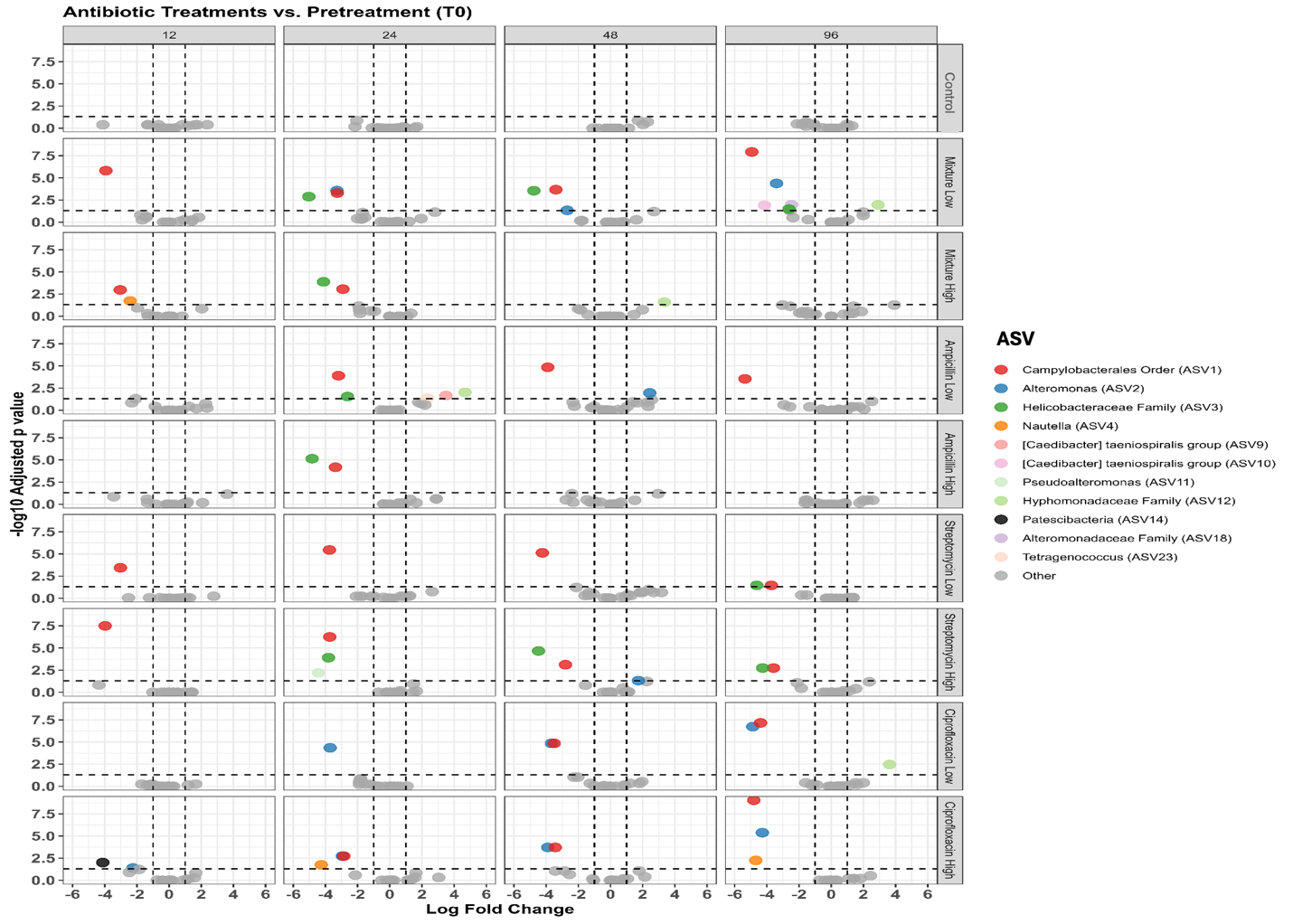


**Fig. S6** Volcano plot depicting differentially abundant taxa by treatment over time, as determined by ANCOM-BC2. Each treatment group was compared against the pretreatment samples (T0) from each specific group. Taxa below the horizontal dotted line are non-significant.


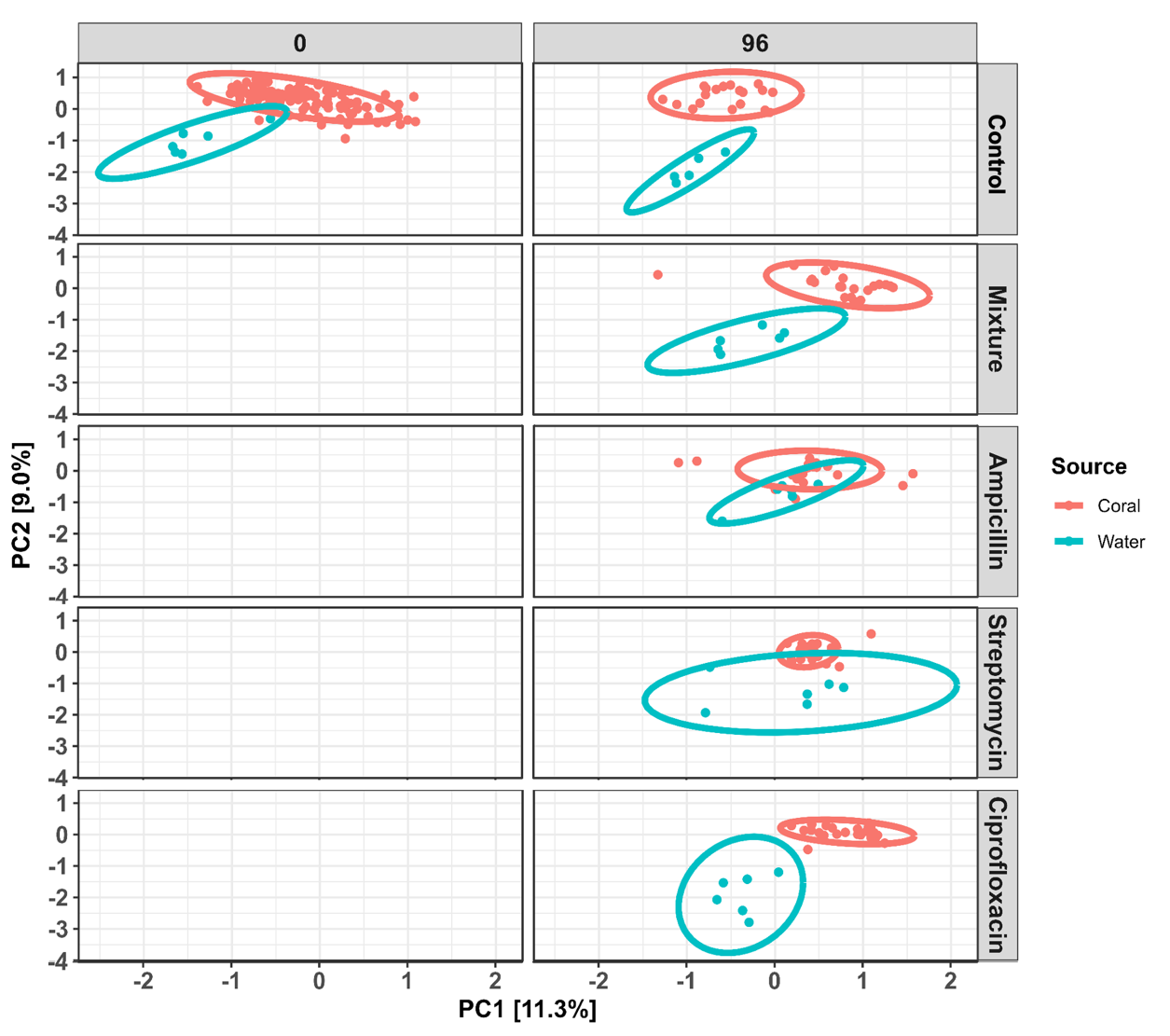


**Fig. S7** PCA displaying dissimilarity between coral and water samples by each antibiotic group at time 96. Given the sampling scheme for water, only blank samples have a corresponding time 0.

**
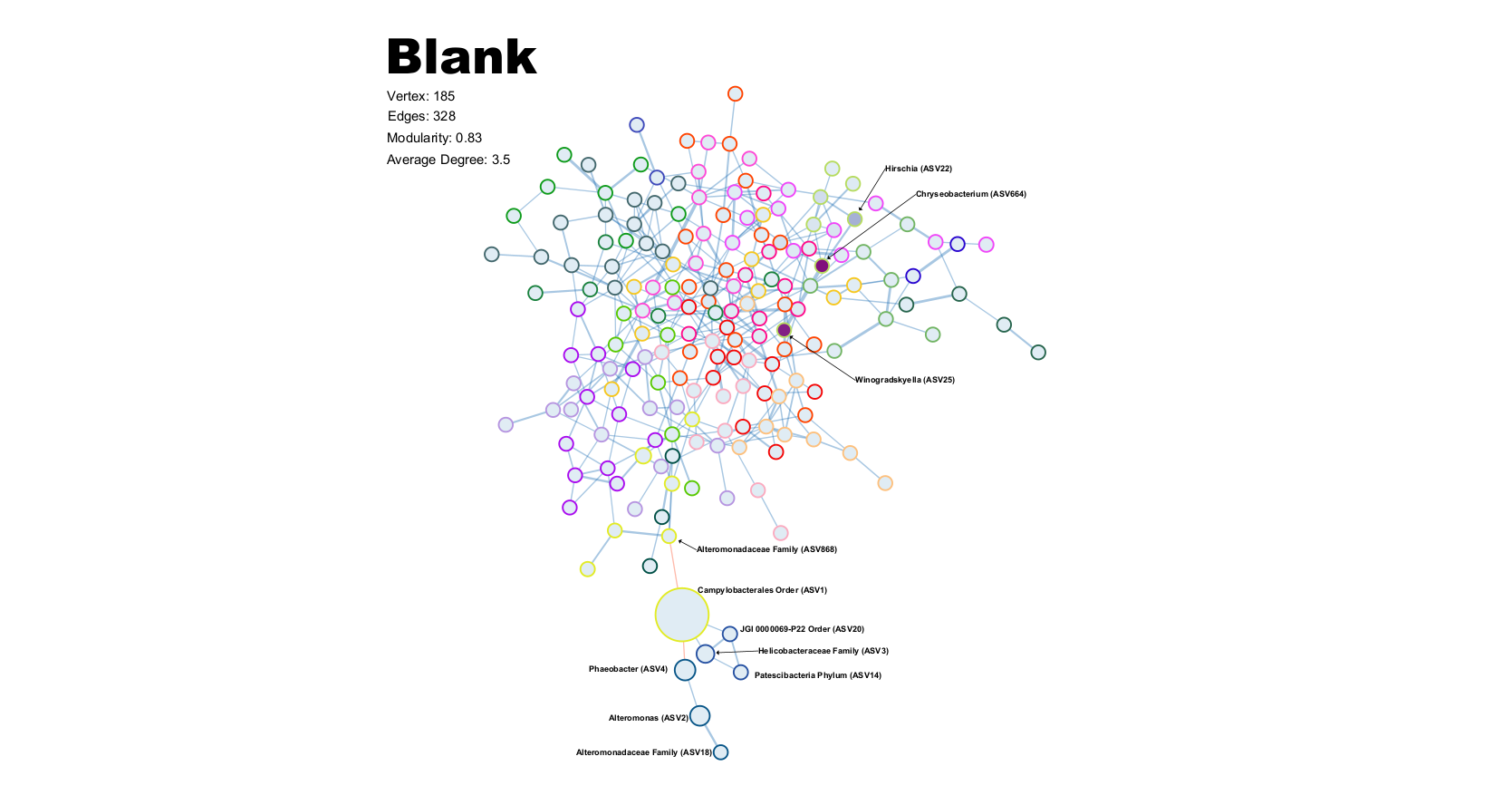

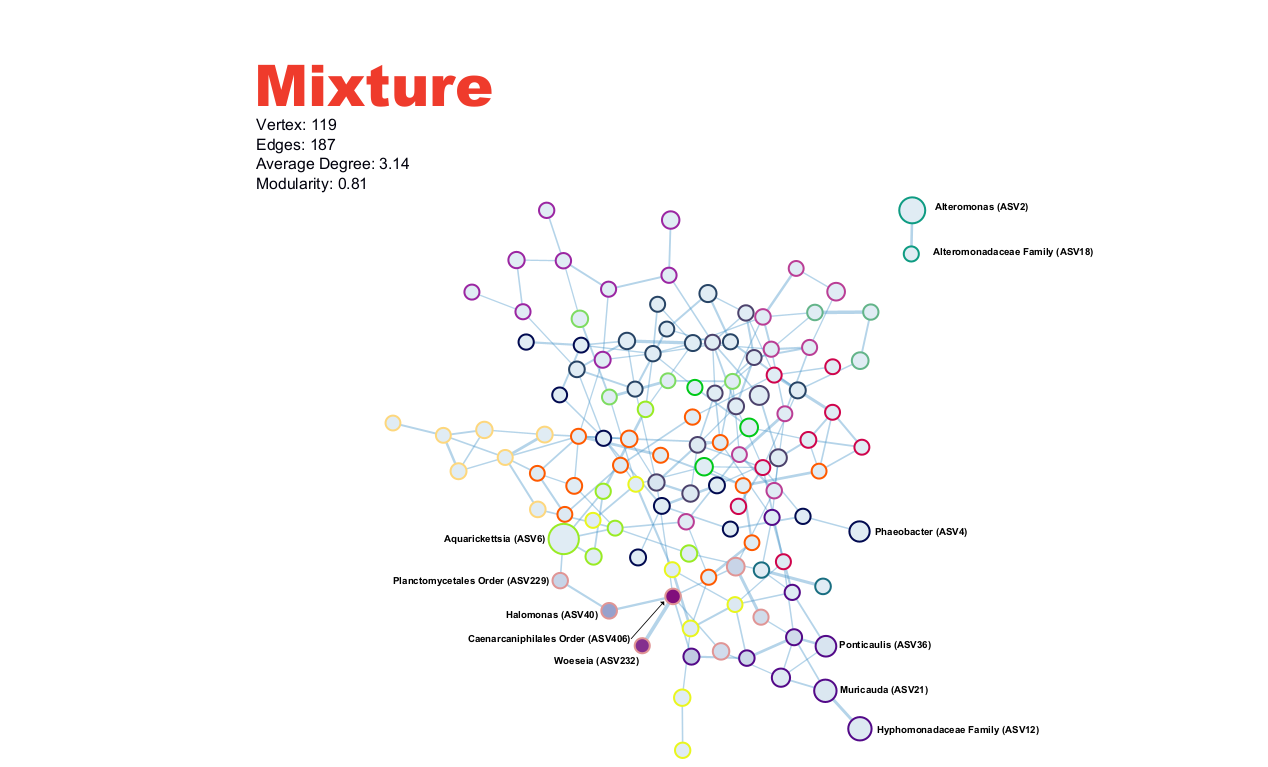
**

**Control**

**
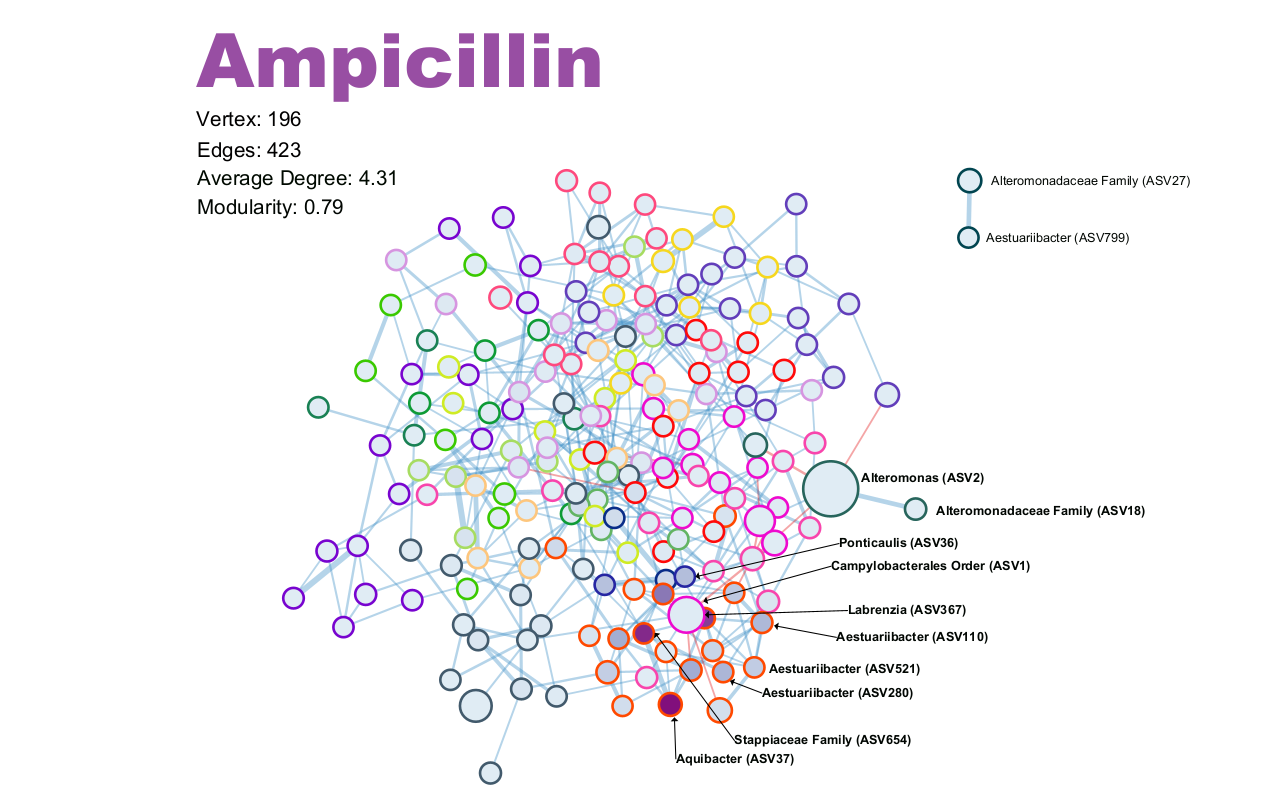

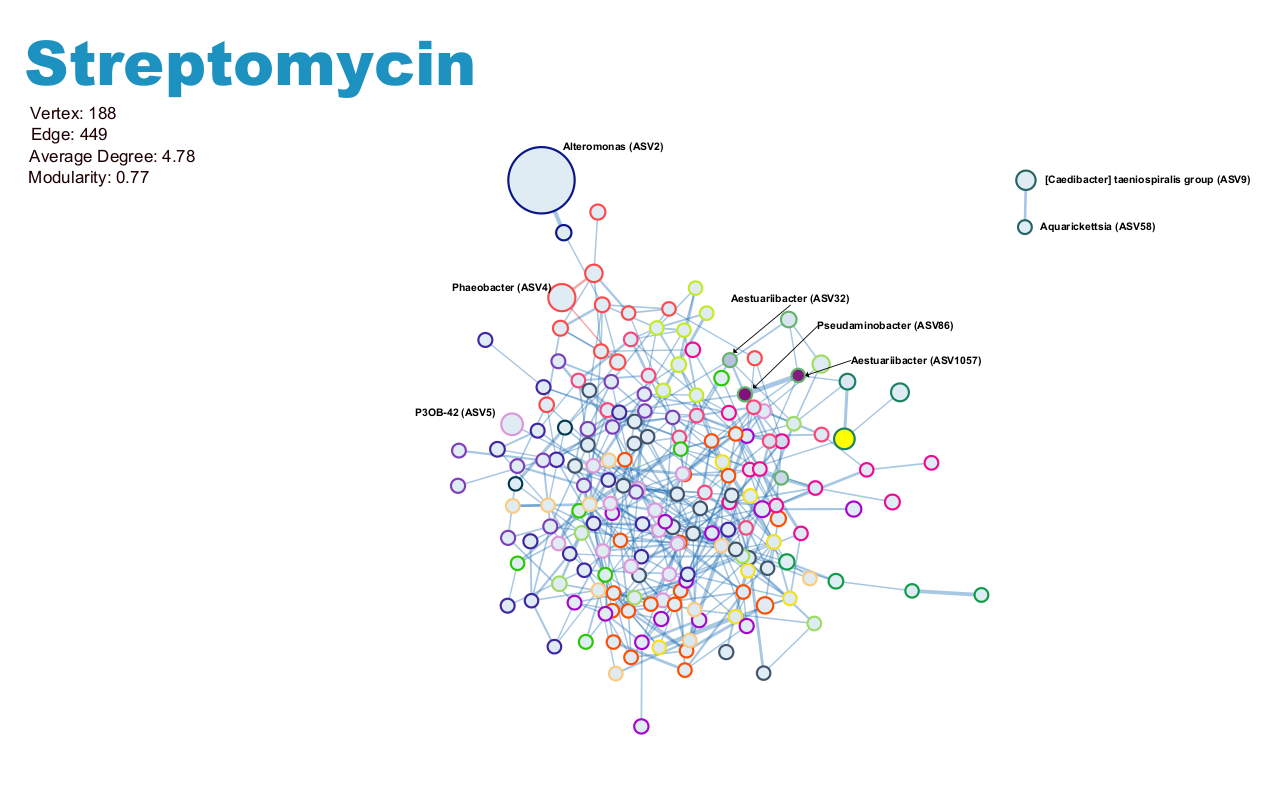
**

**
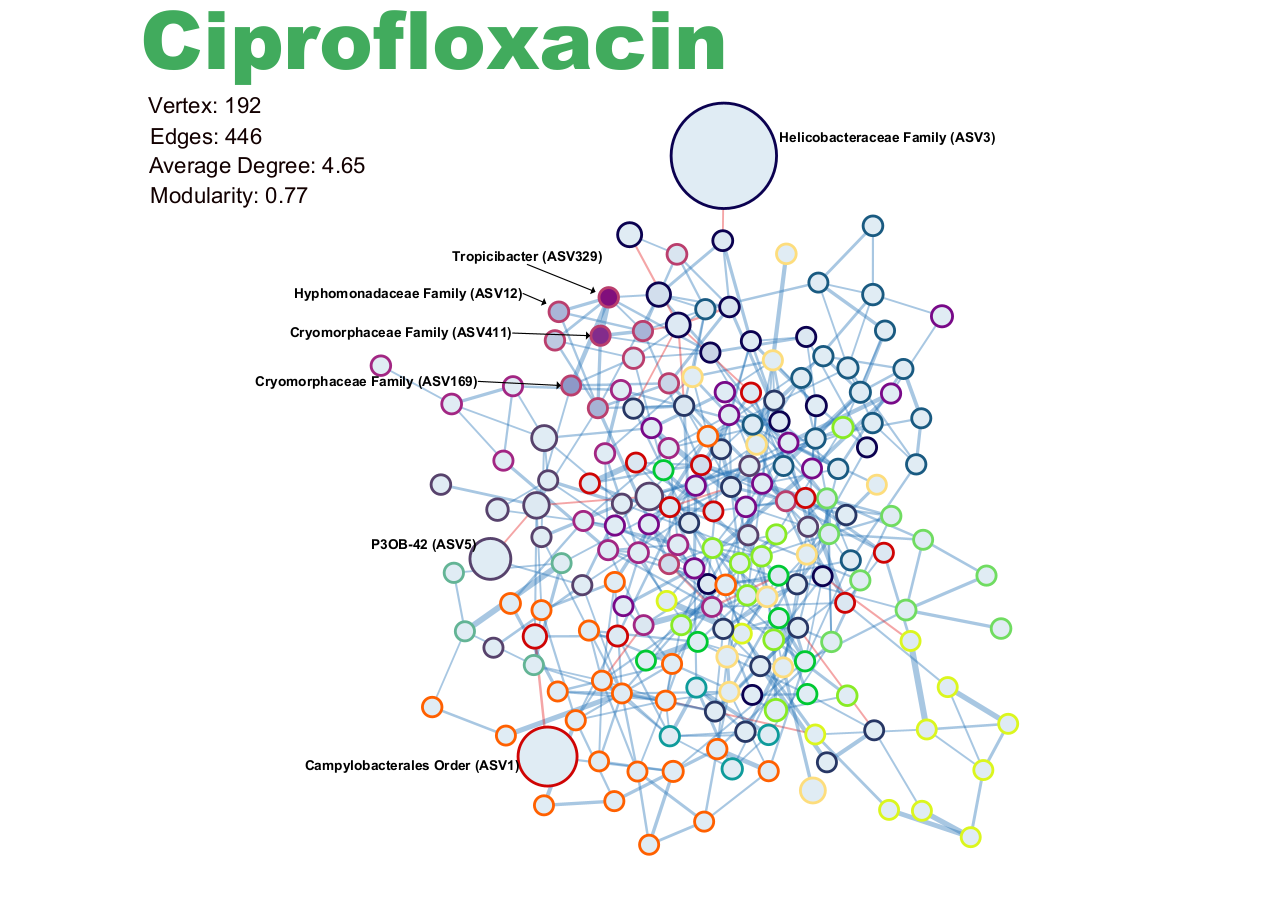
**

**Fig. S8** Networks generated in Cytoscape using node and edge properties calculated from the SPIEC-EASI method. Nodes are filled on a continuous scale based on eigenvector values, with darker colors representing higher eigenvector values. Node edge colors represent different modules, although modules are not consistent between networks. Node size is scaled by relative abundance. Edges are colored by positive (blue) or negative (red) interactions, and the thickness of the line represents the strength of the interaction.

**
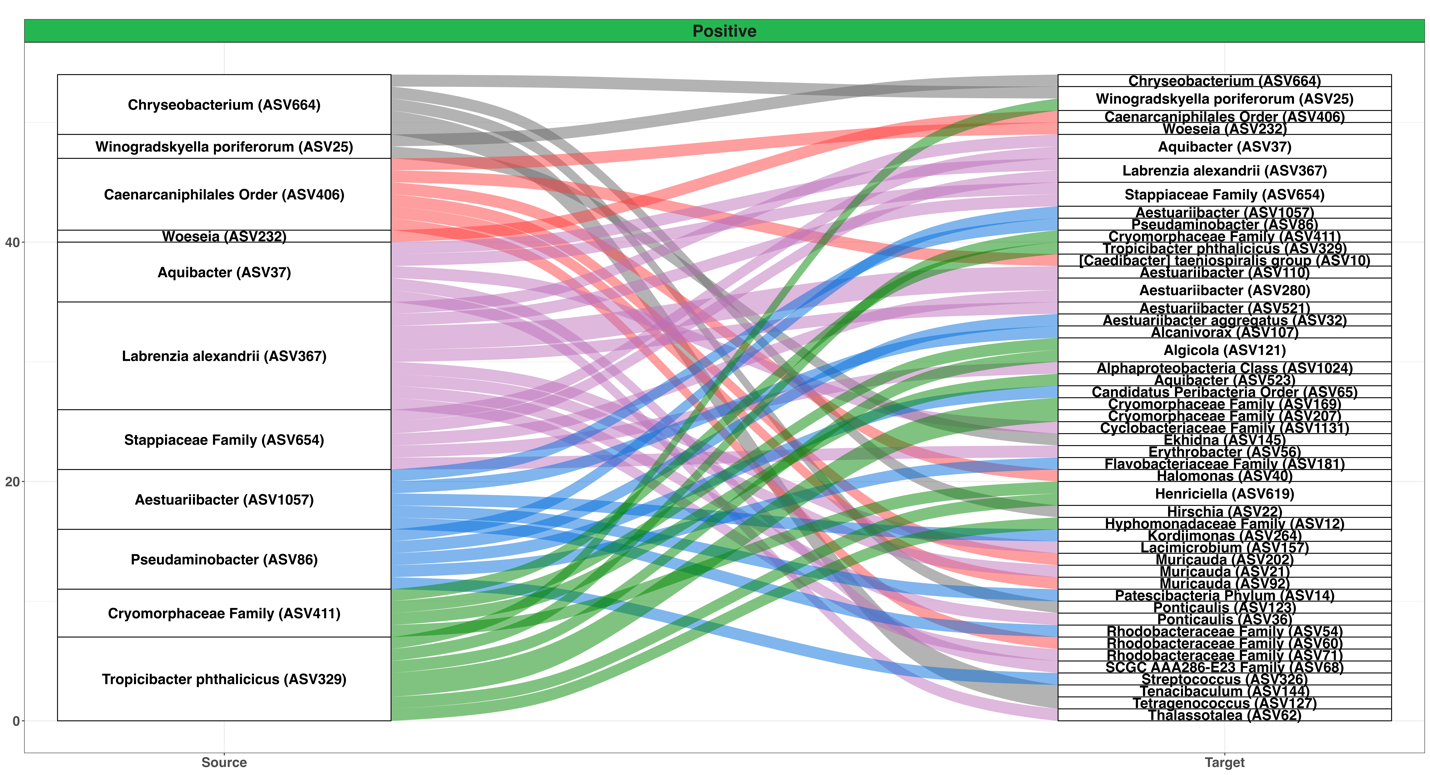
**

**Fig. S9** Alluvial plot displaying the positive microbial co-occurrence patterns between network hub nodes. Interactions within each network are indicated by colors (control is grey, mixture is red, ampicillin is purple, streptomycin is blue, and ciprofloxacin is green). Co-occurrence patterns were determined using SpiecEasi, and no negative interactions were observed.

**Supplementary methods**

*Antibiotic preparation and dosing*

Antibiotics were added slowly to filter-sterilized seawater taken from the CAOS header tank output, and mixed thoroughly. If antibiotics were not dissolving easily, the solution was gently heated using a water bath. Concentrated stock solutions (120 g/L) of ampicillin and streptomycin were made by adding 12 g of each antibiotic to separate sterilized bottles, and adding 0.2 µm filter-sterilized seawater up to 100 mL. 15 mL aliquots of high-dose working solutions (40 g/L) of these two antibiotics were made by diluting 5 mL of 120 g/L solution in 10 mL of filter-sterilized seawater. 15 mL aliquots of low-dose working solutions (4 g/L) were made by diluting 1.5 mL of the 40 g/L solution in 13.5 mL of seawater. Ciprofloxacin solutions were made in the same manner, except the concentrated stock solution was 12 g/L, the high-dose working solution was 4 g/L, and the low-dose solution was 0.4 g/L. To prepare the antibiotic mixtures, 5 mL of each antibiotic stock solution (120 g/L for streptomycin and ampicillin, and 12 g/L for ciprofloxacin), was combined to create the high-dose working solutions, then the low-dose working solutions were made in the same manner as previously described. These 15 mL aliquots were added to the appropriate tanks following the first sampling time point such that the 15 mL antibiotic solution would make the final concentration in the 6L of tank water 100 mg/L (100 µg/mL) for high-dose streptomycin and ampicillin, 10 mg/L (10 µg/mL) for low-dose streptomycin, low-dose ampicillin, .and high-dose ciprofloxacin, and 1 mg/L (1 µg/mL) for low-dose ciprofloxacin. High-dose mixture tanks contained 100 mg/L of streptomycin and ampicillin, and 10 mg/L of ciprofloxacin. Low-dose mixture tanks contained 10 mg/L of streptomycin and ampicillin, and 1 mg/L of ciprofloxacin. At all time points after time 0, partial water changes (3 L) were removed and replaced with fresh seawater from the header tank system. To ensure the antibiotic concentration remained consistent over the antibiotic exposure, 7.5 mL of these high- and low-dose solutions were added to the tanks instead of the original 15 mL. At each time point, blank tanks were supplemented with equal volumes of filter-sterilized seawater that was used to make the antibiotic solutions.

*Raw read quality control and sequence preprocessing*

Low-quality sequences were edited and filtered using several steps: 1) First, to remove low-quality bases, reads were truncated at the 3’ end at 245 bp and 230 bp for the forward and reverse reads, respectively, based on MultiQC reports. 2) Then low-quality reads were removed when a quality score of 2 was identified. 3) Then maximum expected error was calculated and any reads that exceeded a maximum error rate of 2 were also removed.

*Removing sequence contaminants*

Contaminant taxa were identified through the combined method in the decontam package, such that samples were manually designated as either a true sample or a negative control, and corresponding DNA quantification values were provided for all samples — for both true samples and negative controls. The threshold used was 0.5 instead of the default 0.1 to ensure parameters were not overly permissive.
